# Active Pectin/Carboxymethylcellulose Composite Films for Bread Packaging

**DOI:** 10.3390/molecules30112257

**Published:** 2025-05-22

**Authors:** Lavinia Doveri, Yuri Antonio Diaz Fernandez, Giacomo Dacarro, Pietro Grisoli, Chiara Milanese, Maria Urena, Nicolas Sok, Thomas Karbowiak, Piersandro Pallavicini

**Affiliations:** 1 Department of Chemistry, University of Pavia, Via Taramelli 12, I-27100 Pavia, Italy; ydf@unipv.it (Y.A.D.F.); giacomo.dacarro@unipv.it (G.D.); chiara.milanese@unipv.it (C.M.); 2 Department of Drug Science, University of Pavia, Via Taramelli 12, I-27100 Pavia, Italy; pietro.grisoli@unipv.it; 3 Institut Agro, Université Bourgogne Europe, INRAE, UMR PAM, F-21000 Dijon, France; maria-fernanda.urena-vega@agrosupdijon.fr (M.U.); nicolas.sok@agrosupdijon.fr (N.S.); thomas.karbowiak@agrosupdijon.fr (T.K.)

**Keywords:** active packaging, zeolites, pectin, carboxymethyl cellulose, silver

## Abstract

A new active composite film intended for bread packaging is described here. The active film has the aim of prolonging bread’s shelf life while avoiding the use of nanoparticles that, with very few exceptions, are a type of material not allowed by regulatory agencies like EFSA (European Food Safety Agency) and FDA (US Food and Drug Administration) in food contact materials. Moreover, the increasing consumer demand for natural and wholesome products, possibly “clean label”, and packaged in natural, non-petroleum-based materials has been taken into consideration. Accordingly, precursor materials from renewable sources were used to prepare the active film: pectin from citrus peel (PEC) and carboxymethyl cellulose (CMC) were used as the matrix, with oleic acid (OA) as plasticizer. Moreover, the bread preservative calcium propionate (CaP) was used as the crosslinker, and also zeolite microparticles loaded with silver ions (AgZ) were added to the films as an additional antimold agent. This strategy allows us to avoid the addition to bread of the now commonly used preservatives ethanol and calcium propionate, moving the latter to the packaging. Permeance measurements revealed excellent barrier properties against O_2_ and CO_2_, while the typical high water vapor permeance of polysaccharide films was mitigated by the non-hydrophilic OA plasticizer. Moreover, the quantities of Ag^+^ and CaP released in bread are low and below the limits imposed by regulatory agencies. The antimold activity of the films is excellent, with *Aspergillus niger*, *Penicillium janthinellum*, and *wild-type Penicillim* molds reduction on bread in the 99.20–99.95% range for the films containing only CaP and in the 99.97–99.998% range for the films containing both CaP and AgZ. Finally, the rheological properties of the film-forming solutions were investigated, demonstrating their potential application as coatings on natural packaging materials for bread, such as paper.

## 1. Introduction

Despite the huge advantages of using polyolefin films as packaging for food, when the collection and recycling systems fail, they become a source of waste and pollution. In the European Union, the problem of plastic waste and pollution is gaining more attention. Recycling has received a huge boost [1], and various strategies have been implemented to reduce the production of petroleum-based plastic [2]. Moreover, although on a still uncertain time scale, it is important to note that natural oil reserves are destined to run out.

An obvious solution suitable both to mitigate the plastic waste and pollution problem inherent to food packaging, and to serve as an alternative for petroleum-derived packaging films, is to use polymers derived from renewable sources. Polysaccharides are among the best choices for producing biodegradable and/or compostable packaging [3]. Polysaccharides have many favorable features: they are non-toxic and biodegradable, they display good film-forming ability [3,4], they have good oxygen barrier properties, and they have acceptable mechanical properties when compared to petroleum-based plastics [5]. On the other hand, a drawback in the use of polysaccharide films for food packaging is their hydrophilicity [3,6], which has suggested their use as coatings rather than self-standing films, e.g., in paper-based or multilayer packaging [6,7].

Bread matrix has a relatively high moisture content and a water activity between 0.94 and 0.97, with a pH of about 6, so it is a favorable environment for the germination and growth of molds and bacteria [8,9,10]. While bread is perfectly sterile when it comes out of the oven, when it is cooled, sliced, packed, and wrapped, it undergoes microbial contamination. Polyethylene (PE) films are commonly used for bread packaging, with chemicals added to the packed bread to extend its shelf life. Calcium propionate (CaP) and/or potassium sorbate (KS) are added to bread during its preparation to inhibit the growth of microorganisms after baking [8,10]. Quantitative limitations hold depending on the country. In Europe, the maximum CaP allowed is 0.2% (*w*/*w*) and 0.3% (*w*/*w*) KS in prepacked rye bread, and 0.1% (*w*/*w*) for CaP in prepacked, unsliced bread [11]. While such percentage values seem small, it should be stressed that by eating a single bread portion, with a typical 50 g mass, a consumer ingests from 50 to 150 mg of preservatives. Another widely used preservative is ethanol, which is directly sprayed on bread before packing. An amount of ethanol ranging from 0.2% to 12% *w*/*w* is common, without restrictions in the EU [8], sometimes in combination with KS or CaP [12]. Physical sterilization methods are also possible, such as UV or IR irradiation and microwave heating, but they are very rarely used in bakeries because of their high cost and low efficiency [8,10].

High concentrations of chemical preservatives affect the taste and the sensory properties of bread [10], and the prolonged use of them may lead to fungal resistance [13]. In addition, the consumers demand for baked food has recently shifted towards natural and wholesome products, possibly “clean label”, i.e., without added chemical preservatives [10]. As regards bread, this presents a highly demanding challenge, because bread packed in PE [14] without preservatives has a shelf life of only a few days at room temperature [10]. Active packaging has been put forward to preserve food and to meet the demand of consumers towards “clean label” foods. Active materials are defined as materials intended to extend the shelf life or to maintain or improve the condition of packaged food by releasing or absorbing substances [15]. In particular, an extension of the shelf life of bread has been proposed by modifying the common PE packaging with embedded micro- or nanoparticles, either for the release of small, controlled quantities of active substances [8,14] or for the improvement of the oxygen barrier properties of the packaging to prevent food oxidation [14,16]. Active packaging has also been implemented in films for food packaging made of polysaccharides [17]. Interestingly, for the goals of the present research, only a few of the reported examples were studied for bread packaging [18]. Most researchers have proposed to embed silver nanoparticles (AgNPs) in the packaging film as the easiest way to develop active packaging with antimold and antibacterial action [19,20,21]. AgNPs are well known as an extremely active material against bacteria and molds, thanks to the sustained release of the Ag^+^ cation, which is the actual antimicrobial agent [22]. Unfortunately, AgNPs are usually not included by regulatory agencies in the substances allowed in food contact materials; see, e.g., EFSA in the EU [23,24]. Noticeably, the same regulatory agency excludes the use of nanoparticles of any material, with very few exceptions like titanium nitride, ZnO, and nanoclays. Besides nanoparticles, microparticle-embedded natural antimicrobial agents, such as the extract of herbs and spices or essential oils, are also widely explored in the literature [8,14,16]. However, the already stressed disadvantage of food sensory properties modification limits the amount that can be added and consequently the antimicrobial capacity [8].

**Scheme 1:**
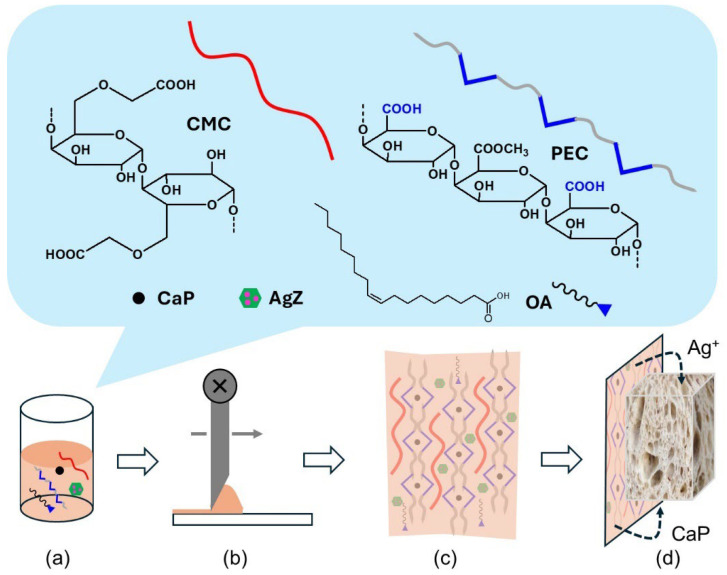
Composition of the active film and stages from film preparation to its use, with (**a**–**d**) corresponding to film precursor solution (**a**), tape casting of the film with doctor blade technique (**b**), self-standing film with all embedded components (**c**), and contact of the film with bread and migration of the active components (**d**). CMC = Carboxy methylcellulose CaP = Calcium propionate, PEC = pectin, OA = oleic acid, AgZ = Ag^+^-loaded zeolites).

Pectin (PEC, formula in Scheme 1) is an anionic polysaccharide made mainly of D-galacturonic acid residues linked with α-1,4-bonds and defined as low methoxyl pectin when the carboxylic groups of uronic acid are partially methyl esterified (DE < 50%) [25]. Its films have a very high barrier to oxygen, reasonably good (i.e., low) water vapor transmission rates [7,26], and also exert an efficient barrier effect for oil and aroma preservation [25]. As a drawback, PEC is hydrophilic and has poor mechanical properties, since its films are brittle and rigid [7,26].

CMC is a water-soluble cellulose derivative made of glucopyranose residues linked by β-1,4 bonds. Thanks to the presence of a hydrophobic polysaccharide backbone and of many hydrophilic carboxyl groups, CMC is less hydrophilic than PEC [27]. Moreover, it has good mechanical properties, as its films are flexible, easily stretched, and ductile. PEC and CMC have already been used for the preparation of single-component biodegradable films [7,28,29,30,31,32,33] and as mixtures [25,27]. In most cases, glycerol was used as a plasticizer agent, improving the mechanical properties of the film but also exerting a negative influence on its barrier properties: an increase in the mesh size of the biopolymer network was observed, and, due to glycerol hydrophilicity, the water vapor transfer also increased [26]. Oleic acid (OA, Scheme 1) was studied as an alternative plasticizer for polysaccharide films, obtaining satisfactory mechanical properties and an improved barrier against water vapor [34].

Ca^2+^ is a cation capable of forming strong bridges between the carboxylic groups of the galacturonic chains of pectin when the pH is below the pKa of the carboxyl groups, with the so-called egg-box model mechanism sketched in Scheme 1c, promoting pectin gelation [35]. Zeolites (Z) are microporous aluminosilicates that are low cost, non-toxic, naturally abundant, and with remarkable ion exchange capacity [36]. Moreover, the addition of Z can improve the mechanical [36,37] and barrier properties of self-standing films [36]. Z can be loaded with metal cations, including, significantly, Ag^+^ [36,37,38]. In this regard, it must be mentioned that the limiting quantity of Ag^+^ allowed in food by EFSA is 0.05 mg Ag/kg food [39].

In this paper, self-standing films are presented, made of PEC and CMC, with CaP as the crosslinking agent, OA as the plasticizer, and with embedded zeolites loaded with Ag^+^ (AgZ). Such films are a new active packaging designed to allow the preservation of bread, avoiding the common practice of adding huge quantities of chemical preservatives, such as ethanol, CaP, or KS, in the bread. To this aim, the CaP antimold preservative was transferred from the bread to its packaging, and an Ag^+^-releasing agent, which is not nanosized, was incorporated into the same packaging. As sketched in Scheme 1b, the traditional solvent casting method [25] was avoided, and the film was prepared with a tape casting “doctor blade” approach that allows control of the thickness homogeneity and avoids the use of diluted film-forming solutions. The sum of all these features is a novelty in active packaging, especially considering the simultaneous double action of CaP and Ag^+^, Scheme 1d. The barrier, mechanical, and optical properties were evaluated on the self-standing films, along with the release of Ag^+^ towards bread, in order to achieve values within the limits imposed by EFSA [39]. The antimold activity and shelf life experiments on bread were performed in order to demonstrate the effectiveness of the studied films with respect to traditional, non-active packaging. Finally, the viscosity of the film-forming solutions was determined to assess their applicability as coatings.

## 2. Results and Discussion

### 2.1. Film Formulation

Preliminary studies were carried out exploring a range of mass ratios of PEC, CMC, OA, and calcium salts for the preparation of the PEC/CMC self-standing films. The quantities of OA (0.30 g) and PEC (3.0 g) were kept constant, while the quantity of CMC was varied from 10% to 40% *w*/*w* (with respect to pectin), and the amount of Ca^2+^ from 0.75 mmol to 1.9 mmol. The mechanical properties of all the prepared films were determined and are reported in SI1. An increase in the tensile strength (TS) was observed on increasing the amount of CMC in the formulation: 34(±8) MPa for 10% CMC, 38(±18) MPa for 20% CMC, and 57(±10) MPa for 30% CMC. The film with the maximum quantity of CMC (40% *w*/*w*) showed instead a decrease in the TS (40(±13) MPa). Elongation at break (E%) was not affected by the different quantity of CMC (constant value ~7–8%). Concerning the amount of crosslinker (Ca^2+^ salt), the rationale was to choose the maximum possible quantity to maintain good TS and E%. These preliminary studies revealed that the films with the best mechanical properties are prepared with 30% *w*/*w* of CMC and 1.9 mmol of Ca^2+^, as higher quantities of Ca^2+^ crosslinker generated cracks in the films. All the studies described in the following sections were conducted with these weight proportions of the components, following the protocol described in detail in Section 3.2.1

### 2.2. Functional Properties of PEC/CMC Self-Standing Films

#### 2.2.1. Mechanical Properties

TS, YM, and E% are critical parameters to be evaluated for the design of food packaging. Among all its functions, packaging also has to maintain its integrity during storage, distribution, processing, and handling [40].

TS indicates the maximum resistance of a film to breaking under tension. YM is correlated to TS and discloses the intrinsic stiffness of the films. The flexibility of the films is instead given by the value of E%. The mechanical properties of PEC/CMC films are displayed in Table 1. The thickness, TS, YM, and E% of Mater-bi^®^ (MB) commercial sachets and of a low-density PE commercial film (LDPE) were also measured and included in Table 1. It has to be stressed that the comparison with the films prepared in this paper has limitations, as the preparation techniques of the examined commercial LDPE and MB films are unknown and most probably different from the tape casting method used in the present paper. However, MB is the most common biodegradable film currently produced and used (e.g., on the market for picking fruits and vegetables), and LDPE is commonly used for sliced bread packaging. The properties of these two films relevant to bread packaging have therefore been measured in this research to allow a reasonable quantitative comparison between the new films described here and the market standards.

The examined MB is thinner (12.6(5) µm) but of comparable thickness with the LDPE film (51(1) µm). Using a total mass of solid precursors (PEC, CMC, Ca salts, OA, Z, or AgZ) ranging from 4.3 to 4.8 g (depending on the chosen Ca salt and on added/not added Z or AgZ), two films of approximately 12 × 20 cm^2^ were obtained with the “doctor blade” technique (see SI2). The thickness of the films prepared in this study, Table 1, is in the 40–65 µm range, depending on the composition. Moreover, the low standard deviation indicates that the “doctor blade” technique yields homogeneous and reproducible films, with a thickness almost identical to that of the LDPE used for bread packaging. PEC/CMC films are more resistant than the commercial MB and LDPE used for comparison, displaying a higher TS, a feature that is optimal for films intended for food packaging. Moreover, the addition of a crosslinker further enhances the TS, stepping from 35(±5) MPa of PEC/CMC/OA to 50–70 MPa of the films with Ca^2+^ ions. On the other hand, the flexibility of LDPE and MB is, respectively, three and two orders of magnitude higher than that of our films, although Ca^2+^ also positively affects the E%, which increases from 2.6% (no Ca^2+^) to 7–9%. The YM decreases significantly with the addition of Ca^2+^, i.e., leading to less rigid films, with YM values comprised between those of MB and LDPE. The use of Ca^2+^ as a crosslinker thus contributes positively to the mechanical properties of the films, as it improves their elasticity and strength while reducing the stiffness. Moreover, the literature shows that the mechanical properties of the films described in the present paper are close to those of other petroleum-based plastics of current use: e.g., TS has been reported in the 8–20, 30–40, and 48–72 MPa ranges for LDPE, PP, and PET, respectively [41]. As for zeolites (with or without silver), their addition does not significantly affect the mechanical properties of the films. Finally, comparison with pectin films without CMC (SI3, Table) highlights the already recognized brittleness of PEC-only films [7,26], which have much lower TS and E% values and a higher YM. The addition of CMC can thus be considered beneficial from a mechanical point of view.

#### 2.2.2. Barrier Properties

Mass transport properties of films are crucial parameters to evaluate their performances and determine their effectiveness for food packaging. Water vapor, oxygen, and carbon dioxide can penetrate through the packaging film or, in the case of humid food like bread, leave the sachet during storage.

**Figure 1:**
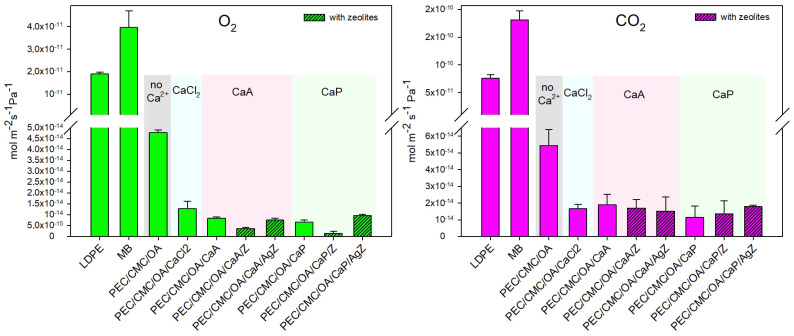
Oxygen (**left**) and carbon dioxide (**right**) permeance of composite films, compared with LDPE and MB. Bars for films containing zeolites are represented with a line pattern, and background colors guide the eye to the Ca^2+^ salt used as a crosslinker. All data were measured at 25 °C, RH = 0%. Each value is the average of five measurements. Error bars (standard deviation) are displayed in the graph. Numerical values and statistical analysis are reported in SI4.

All this affects the microbial growth, the product texture, the quality of food (e.g., by oxidation), and sensory features [42]. It has been shown that staling, spoilage, and lipid oxidation of bread are promoted mainly by the presence of oxygen inside the packaging [8,14,42]. CO_2_ could instead play a favorable role, as it can be added in the packaging sachet to suppress or limit microbial growth in bread [14,42]. Figure 1 displays the O_2_ and CO_2_ permeance of the composite PEC/CMC films and of LDPE and MB. The O_2_ barrier performance of the PEC/CMC films is excellent. The O_2_ permeance of all the PEC/CMC composite films with added Ca^2+^ salts is in the 1–9 × 10^−15^ mol m^−2^s^−1^Pa^−1^ range, which is four orders of magnitude lower than that of the commercial LDPE (pO_2_ = 1.90(±9) × 10^−11^ mol m^−2^s^−1^Pa^−1^) and MB (pO_2_ = 4.0(±7) × 10^−11^ mol m^−2^s^−1^Pa^−1^) used for comparison. This is not unexpected, as the literature reports high O_2_ barrier values for PEC films, with reported permeabilities as low as 6.75 × 10^−15^ mol m^−2^s^−1^Pa^−1^ [43]. In the case of LDPE and MB, a structure without or with poor polar interactions and/or hydrogen bonds has affinity for the non-polar molecule dioxygen [44], thus promoting permeation. The O_2_ barrier properties of biodegradable and non-biodegradable polymers at RH = 0% have been classified by Wu et al. [45]. According to this classification, all PEC/CMC films with CaA and CaP belong to the “very high barrier to oxygen” group (permeance < 7.58 × 10^−15^ mol m^−2^s^−1^Pa^−1^), and the PEC/CMC film with CaCl_2_ as crosslinker fits into the “high barrier” group (permeance between 7.58 × 10^−15^ and 7.58 × 10^−14^ mol m^−2^s^−1^Pa^−1^), while LDPE and MB are classified in the “poor barrier” group (permeance > 7.58 × 10^−12^ mol m^−2^s^−1^Pa^−1^). Adding zeolites to PEC/CMC leads to an even lower O_2_ permeance, an effect that is reasonably connected to the known increase in the tortuosity pathway of various gases when nano- or microparticles are added as fillers to polymeric films [46,47,48]. It has to be stressed that changing CaCl_2_ with CaA or CaP has a beneficial effect (i.e., decrease) on O_2_ permeance. As a result, the PEC/CMC/OA/CaP/Z film is the least permeable to oxygen (pO_2_ = 1.4 × 10^−15^ mol m^−2^s^−1^Pa^−1^). Interestingly, the addition of CMC enhances the O_2_ barrier properties with respect to PEC-only films (SI5, Table), although cellulose derivatives are usually more permeable to the same gas [7,26]. Moreover, PEC-only films also show a decrease in O_2_ permeance on changing the crosslinker from CaCl_2_ to CaA and CaP. A rationalization of the differences in O_2_ permeance can be put forward by observing the pH values of the film-forming solutions listed in Table 2 (see also SI6, Table) and the data in Figure 1 left (O_2_ permeance). The overall trend observed here is that the higher the pH of the film-forming solution, the better the O_2_ barrier properties of the obtained films.

The pKa of PEC is 3.5 [49], and the pKa of CMC is 4.4 [50]. With a 3.5 pKa value, increasing the pH in the 3.5–5.00 range increases the % of deprotonated carboxylic groups of PEC (and CMC) leading to a more effective “egg-box” crosslinking due to the coordination of the -COO^−^ groups of PEC to the Ca^2+^ cation. As a consequence, the structure of the film is denser, and it is more difficult for O_2_ to penetrate and cross the films. The pH of the film-forming solutions decreases with the addition of CaCl_2_ (3.89→3.50), due to the weak Lewis acid character of the Ca^2+^ cation but increases with CaA (4.40) and CaP (4.80), thanks to the weak base nature of the acetate and propionate anions. Also, the addition of zeolites helps to increase the pH, adding a contribution to lower O_2_ permeance, besides their intrinsic role in increasing the tortuosity pattern of gas diffusion.

The nature of the permeants (their molecular volume and affinity to the polymer matrix) affects the mass transfer through the films. CO_2_ is a molecule larger than O_2_; it has a lower diffusivity coefficient but a higher solubility coefficient with respect to oxygen [41]. Figure 1 shows the CO_2_ permeance of all composite films compared to that of LDPE and MB. Also, in this case, the permeance of CMC/PEC films is 3–4 orders of magnitude lower than that of LDPE and MB, and a significant decrease is observed with the presence of Ca^2+^ salts as crosslinkers compared to the PEC/CMC/OA film not crosslinked. However, in the case of CO_2_, there is no definite trend in changing the Ca^2+^ salt or adding zeolites, as the higher standard deviations found in all data lead to differences that are not significant (*p* < 0.5) from the statistical point of view (SI4, Table).

**Figure 2:**
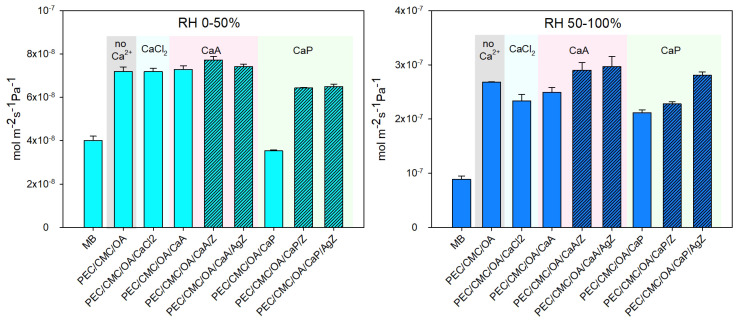
**Left**: water vapor permeance in semi-dry conditions (RH 0–50%). **Right**: water vapor permeance in humid conditions (RH 50–100%). Bars for films with zeolites are evidence with a lined pattern; background colors guide the eye to the Ca^2+^ salt used as a crosslinker. Data recorded at 25 °C. Each value is the average of four samples. Error bars (standard deviation) are displayed in the graph. Numerical values and statistical analysis are reported in SI4.

Water vapor permeance is a fundamental parameter in packaging applications, especially for a fresh, semi-humid food such as bread [18,42]. The water vapor permeance of the films was measured in semi-dry and humid conditions. The corresponding results are shown in Figure 2 and listed in detail in SI4. As a general observation, on passing from humid to semi-dry conditions, the water vapor permeance decreases of approximately one order of magnitude, i.e., from the 10^−7^ to the 10^−8^ mol m^−2^s^−1^Pa^−1^ range. The performance of all the examined films as a barrier to water vapor is not very satisfactory and is worse than that of MB. Only in semi-dry conditions are pH_2_O for MB and PEC/CMC/OA/CaP similar (4.0(±2)10^−8^ mol m^−2^s^−1^Pa^−1^ and 3.53(±4)10^−8^ mol m^−2^s^−1^Pa^−1^, respectively) and belong to the “medium barrier” group [45]. Comparison with the permeance found for LDPE (1.85(±5)10^−9^ and 1.39(5)10^−9^ mol m^−2^s^−1^Pa^−1^ in semi-dry and humid conditions, respectively) evidences the high permeance to water vapor of the films. This is obviously due to the hydrophilicity of polysaccharide-based materials [7] that is only partially mitigated in the present films by the use of OA as the plasticizer. When Z or AgZ are embedded in the films, we observe an increase in the water vapor permeance. The high affinity of Z for water [36] promotes adsorption and transport through the film.

#### 2.2.3. Morphological Characterization

**Figure 3:**
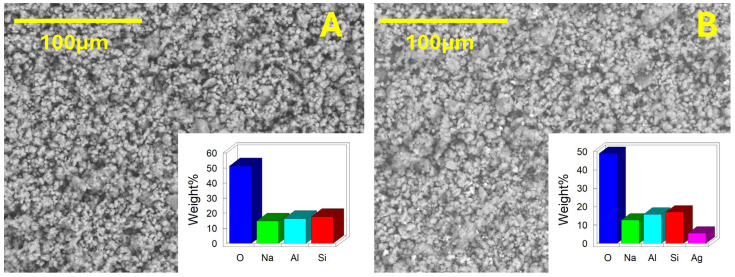
SEM-EDS image of zeolites (**A**) and silver-treated zeolite (**B**) powders; the insets show the % weight elemental composition, determined by SEM-EDS.

Morphological and chemical analyses of the used zeolites were carried out by SEM-EDS. Figure 3 shows a homogenous grain size distribution before (A) and after (B) the treatment with silver nitrate. The grain dimensions are in the range of micrometers. The silver-loaded zeolites were prepared by suspending plain Z in an aqueous AgNO_3_ solution, with 5% Ag mass with respect to Z.

Zeolites are sodium aluminum silicates, and their SEM-EDS analysis shows the expected presence of O, Na, Al, and Si (Figure 3A, inset). In the case of AgZ, SEM-EDS analysis also displays the additional presence of Ag at 5.4(±2)% *w*/*w*. Control experiments carried out with 10% and 20% *w*/*w* Ag/Z solutions showed 10(±1)% and 19(1)% *w*/*w* Ag, respectively, in AgZ at SEM-EDS analysis (SI7). This indicates that the used procedure gives AgZ solid materials with the same Ag/Z mass ratio as in solution over a large mass ratio range, allowing a fine tuning of the loaded Ag quantity.

**Figure 4:**
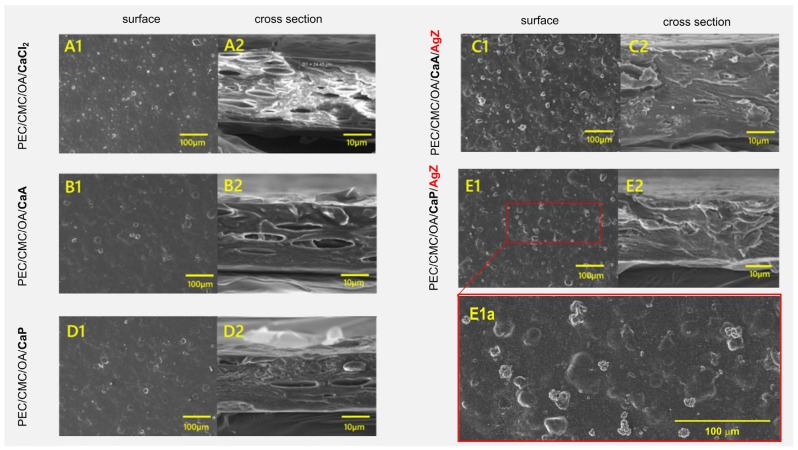
SEM image of the surface (1) and cross-section (2) of pectin/CMC films. (**A1**,**A2**) PEC/CMC/OA/CaCl_2_; (**B1**,**B2**) PEC/CMC/OA/CaA; (**C1**,**C2**) PEC/CMC/OA/CaA/AgZ; (**D1**,**D2**) PEC/CMC/OA/CaP; (**E1,E1a,E2**) PEC/CMC/OA/CaP/AgZ. The film composition, the surface or cross-sectional typology, and the presence of AgZ are also indicated alongside the images, for the sake of clarity. (**E1a**) is a blowup of a section of (**E1**) (delimited by the red dashed rectangle) to evidence the presence of zeolite particles on the film surface. Larger images are available in SI8.

The surface (1) and cross-section (2) of PEC/CMC films were studied using SEM. Images are shown in Figure 4 (see SI8 for larger images). The surfaces of the PEC/CMC/OA/CaCl_2_, PEC/CMC/OA/CaA, and PEC/CMC/OA/CaP films that do not contain zeolites (A1, B1, and D1) are homogeneous except for a few defects, i.e., bubble-shaped voids. Such bubble-shaped voids are more evident in the cross-sections (Figure 4(A2,B2,D2)). While no difference is brought by the three different counter anions, the Ca^2+^ crosslinker is responsible for such voids. Ca^2+^ bridges the negative chains of pectin by -COO^−^ coordination, with the “egg-box” mechanism, and free volume is formed [51]. However, the O_2_ transport experiments have demonstrated that such voids are non-communicating, as the addition of a Ca^2+^ crosslinker actually decreases the O_2_ permeance. When zeolites are added (Figure 4(C1,E1)), microparticles are observed on the film’s surface. Cross-section imaging shows that the bubble-shaped voids disappear (Figure 4(C2,E2)). The zeolites are thus homogeneously dispersed in the films, exerting their known filler action [36], giving more homogeneous and compact materials with generally improved barrier properties (Figure 1), and slightly decreasing the stiffness of the films, as can be seen by comparing the YM of PEC/CMC/OA/CaA and PEC/CMC/OA/CaP with that of the same films containing Z or AgZ (Table 1). The SEM-EDS technique was used to determine the quantity of silver by mapping the area of the AgZ-containing films. The quantity of silver determined was 0.47(3)% *w*/*w* for PEC/CMC/OA/CaA/AgZ and 0.49(3)% wt for PEC/CMC/OA/CaP/AgZ.

#### 2.2.4. Antimold Activity

**Figure 5:**
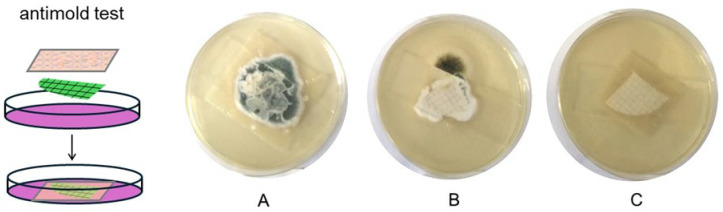
**Left**, sketch of the setup for the antimicrobial tests: a cellulose acetate membrane inoculated with molds (green, squared texture) is placed on a Petri dish with culture medium (purple) and covered with a film portion; (**A**–**C**) membranes (quarter of circle, white, squared texture) inoculated with *P. janthinellum* after 96 h in a Petri dish and coated with PEC/CMC/OA/CaCl_2_ (**A**), PEC/CMC/OA/CaP (**B**), and PEC/CMC/OA/CaP/AgZ (**C**) films; a slide for microscopy was placed on top to assure contact.

The target of this research was to obtain an active polysaccharide packaging film capable of prolonging the shelf life of bread. Accordingly, the antimold activity of the films was tested against the two most common molds formed in bread spoilage, *Penicillium janthinellum* and *Aspergillus niger* [8]. Moreover, a wild-type *Penicillium* mold was used for the same test. This fungal strain was isolated as an indoor contaminant of a grain mill storage [52]. Fungal suspensions were inoculated on cellulose acetate filter membranes and put in contact with PEC/CMC/OA/CaP and PEC/CMC/OA/CaP/AgZ films, as sketched in Figure 5 and described in the Methods section. The PEC/CMC/OA/CaCl_2_, film, which does not contain antimold substances, was used as the control.

A total of 96 h was chosen as the contact time because fungi viability is typically noticeable after 3–4 days of incubation [53,54]. The antimold activity (ME, microbicidal effect) is reported in Table 3, and is expressed as follows:

ME = log (N_c_/N_d_)

where N_c_ is the number of colony forming units (CFU) found with the control, and N_d_ the number of CFU found in the active films.

CaP is a known bread preservative [10], and Ag^+^ is a broad-spectrum antimicrobial whose antimold action has already been demonstrated [22,37]. On the basis of the film formulations, it can be calculated that the film pieces in contact with the inoculated membrane (4 × 4 cm^2^, 130 mg mass) contain 10.1 mg CaP (PEC/CMC/OA/CaP film) and 9.5 mg CaP plus 0.40 mg Ag (PEC/CMC/OA/CaP/AgZ). As can be seen in Table 3, such small quantities are sufficient to secure a huge mold reduction after 96 h even with just CaP as an active agent (99.9%, 99.8%, and 99.2% reduction for *A. niger*, *P. janthinellum*, and *P. wild type*, respectively). When both CaP and AgZ are present in the film, the mold knockdown after 96 h increases to 99.97% for *A. niger*, reaching a remarkable 99.99% for the two examined *Penicillium* strains (99.998% for *P. janthinellum*). Figure 5 visually displays the appearance of membranes inoculated with *P. janthinellum* after 96 h in a Petri dish, covered with the PEC/CMC/OA/CaCl_2_ control film (A), with the PEC/CMC/OA/CaP film (B), and with the PEC/CMC/OA/CaP/AgZ film (C). While in case A the *P. janthinellum* mold has flourished due to the absence of any antimold agent, in case B (film containing CaP) some mold contaminated the Petri dish and grew outside the film-protected area, with only mold traces visible at the edges of the underlying membrane. In case C, the double action of CaP and Ag^+^ kept the membrane perfectly clean and free of mold.

**Figure 6:**
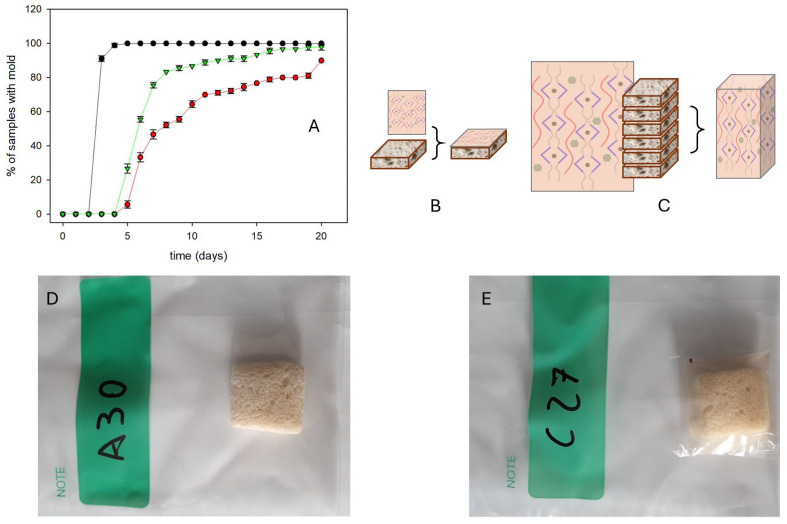
(**A**) Shelf life experiment on bread without preservatives, conducted through visual inspection of samples showing mold traces over time (days) (see the inset for the film used as packaging) (average values on three experiments); (**B**) setup for Ag^+^ release from the film to a single bread slice; (**C**) same setup for a stack of six bread slices; (**D**,**E**) photographs of bread samples (3 × 3 × 1.6 cm) for the shelf life experiments: a bread slice in the LDPE envelope (**D**) and a bread slice wrapped in a PEG/CMC/OA/CaP film inside the LDPE envelope (**E**).

Finally, to qualitatively evaluate the performance of PEC/CMC/OA/CaP and PEC/CMC/OA/CaP/AgZ films as active packaging for bread, shelf life experiments were carried out on freshly produced bread (baked and supplied by Millbo srl, Trecate, Italy) that contained no preservatives. The bread was sliced in 3 × 3 × 1.6 cm^3^ portions. After baking and during the slicing process, the bread was manipulated in an average, non-sterile environment for ~2 h before packaging. Each bread portion was then enveloped in our films and, for comparison, also in LDPE. We enveloped 30 portions of bread for each type of film, using 10 × 6 cm film sections that were externally wrapped in LDPE to avoid further environmental contamination. As shown in Figure 6, such a setup allowed the direct visual examination of the bread surface to search for mold traces. We carried out such visual inspections each day since time 0.

Figure 6A displays the percentage of samples with mold over the total vs. time (days) and the average values for three repetitions. While with the non-active LDPE packaging, mold is developed on 90% of the samples after 2 days and on 100% after 3 days, no mold is visible for the first 3 days with the PEC/CMC/OA/CaP/AgZ and PEC/CMC/OA/CaP films. Moreover, the rate of contamination is lower for the PEC/CMC/OA/CaP/AgZ film than for the PEC/CMC/OA/CaP film, consistent with the ME data listed in Table 3.

#### 2.2.5. Release of Active Species to Bread

A common sliced bread package available in European stores contains 400 g of bread and has 9 × 9 × 22 cm^3^ dimensions. Considering the maximum permitted levels of CaP defined by EC regulations (0.2% *w*/*w* in bread [11]), such an amount of bread is allowed to contain up to 800 mg CaP. The total surface of the packaging film in contact with bread is <900 cm^2^. If one supposes to use the PEC/CMC/OA/CaP films for the same sachet, the total quantity of CaP contained in a 900 cm^2^ film area would be 0.57 g, i.e., even lower than the quantity allowed in the bread. This simple consideration would suggest by itself the safe use of the PEC/CMC/OA/CaP films for bread packaging. However, in view of using this material for bread packaging, it is important to show that the quantity of CaP transferred from the packaging to bread is indeed very low, as this would avoid the ingestion of large CaP quantities by the consumers. An attempt was made to determine the CaP released to 5.0 g bread slices put in contact for 7 days with the PEC/CMC/OA/CaP films. A published method was used [55]; see the Methods section for details. The released CaP was lower than the LOD reported for the method. This does not allow us to determine the exact quantity of CaP released to bread, but it allows us to state that such quantity is lower than 2.0 mg for each bread slice. Noticeably, the maximum EC allowed quantity would be 10 mg in the same sample. On the other hand, the release of Ag^+^ to bread from the PEC/CMC/OA/Ca/AgZ film has to be assessed accurately, as the allowed quantities for this cation in bread are particularly low (e.g., <0.05 mg/kg of bread according to EFSA [39]). The Ag^+^ film-to-bread release test was carried out in two different setups. The first setup is the same used for CaP release (Figure 6B): a 5 g slice of bread (3 × 4 × 1.6 cm^3^) was topped with a 3 × 4 cm^2^ portion of the PEC/CMC/CaP/AgZ film (100 mg, containing 0.31 mg Ag). The second setup (Figure 6C) had the aim to mimic a real sliced bread package: six bread pieces (3 × 4 × 1.6 cm^3^ each, total mass 30.0 g) were enveloped in the PEC/CMC/CaP/AgZ film, with a total contact surface of 158.4 cm^2^ (a film area containing 4.1 mg Ag). Both types of samples were further enveloped in aluminum foil and kept in the dark under controlled 100% humidity, and after 1, 3, and 7 days, the bread was mineralized. ICP-OES analysis followed, and the quantity of silver released to bread was determined. In each test condition, see Table 4, the quantities of Ag^+^ released after 1, 3, and 7 days are very similar, indicating that the film-to-bread Ag^+^ transfer is complete within 24 h of contact. In the case of the stack of bread slices, Figure 6C, the lower and more variable values are to be attributed to the loose contact between the bread and the film envelope, this actually mimicking real shelf conditions. Except for the small exceedance of setup B at 3 days of contact, all the found values are reassuringly equal to or lower than the 0.05 mg/kg limit established by EFSA [39]. While this result is satisfactory, it must also be stressed that the quantity of Ag in the films could be easily fine-tuned to lower values by adjusting either the Ag mass percentage in AgZ or the amount of AgZ added to the film-forming solutions.

#### 2.2.6. Further Characterization in View of Use as Films for Packaging

**Figure 7:**
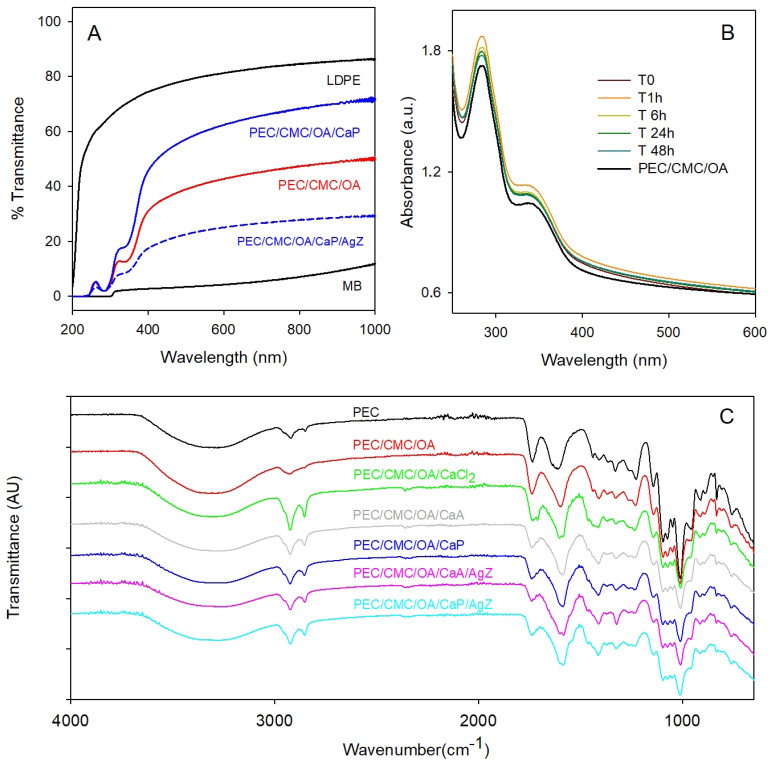
(**A**) Transmission spectra of representative PEC/CMC films, compared with those of LDPE and MB (see the panel for spectra attribution). (**B**) Absorption spectra vs. time of the PEC/CMC/OA/CaP/AgZ under exposure to environmental light; the absorption spectrum of PEC/CMC/OA (black line) is included for comparison. (**C**) FT-IR spectra.

In the FT-IR spectra of the films, the PEC features dominate all the spectra, as PEC is the main component. Neat PEC has a broad absorption around 3300 cm^−1^ (stretching vibration of -OH groups), a three-peak band at 2927 cm^−1^ (stretching vibration of methyl and methylene groups), a band at 1739 cm^−1^ (C=O stretching of the ester group) and a larger one at 1618 cm^−1^ (vibration of the carboxylic acid and carboxylate anion), plus a complex fingerprint envelope at lower wavenumbers [25,27,31]. Such spectral features are maintained in all the prepared films. The 1739 cm^−1^ band decreases in intensity due to the partial hydrolysis of PEC esters during the film preparation, and in the carboxylic acids/carboxylate bands envelope the contribution of -COOH decreases, while the carboxylate band increases (apparent shift to 1618–1583 cm^−1^), mostly due to the PEC-COO- groups coordinated to Ca^2+^ [31]. Slight variations in this spectrum range are observed in the various film formulations due to the diverse contributions of OA, of the acetate and propionate anions, and of the different pH during the synthesis. All spectra are shown in Figure 7C.

Transparency is an additional parameter to be evaluated in food packaging, depending on the packaged food [56]. Also, the UV screen ability of a film must be considered, since UV light may induce photo-oxidation of food or damage photosensitive substances [57]. The transmission spectra of all the composite films were recorded from 250 to 1100 nm (SI9A). The representative spectra of PEC/CMC/OA/CaP and of PEC/CMC/OA/CaP/AgZ are displayed in Figure 7A, together with the transmission spectra of the non-crosslinked PEC/CMC/OA film and of the MB and LDPE films. Transparency is related to the amount of visible light that crosses a film, and it is evaluated by the % transmittance at 550 nm [56]. The crosslinked PEC/CMC/OA/CaP film is more transparent than its non-crosslinked version, PEC/CMC/OA, with T at 550 nm = 52(1)% and 11.3(4)%, respectively, while the addition of Z contributes to the opacity of a given formulation, decreasing T to 21.6(5)% for PEC/CMC/OA/CaP/AgZ. However, as it was already mentioned in describing the shelf life experiment, the transparency in the visible range is enough to actually see the enveloped product and to appreciate its color and texture features. On the other hand, the LDPE transparency is by far superior (T at 550 nm = 80%), while that of the opaque MB is extremely poor (T at 550 nm = 4%). UV screen ability is evaluated by the % transmittance at 280 nm. All the PEC/CMC films are UV-blocking, with a transmittance < 3% at this wavelength, a value one order of magnitude lower than that of the UV–semitransparent LDPE (62.7%) and in agreement with what was already found for pectin-based films [7]. UV–vis absorption spectra were also recorded from 250 to 1100 nm to check the characteristic peaks of pectin and, in the AgZ-containing film, also the eventual formation of nanoparticles during environmental light exposition (promoted by the Ag^+^ to Ag(0) photoinduced reduction). The two characteristic bands of pectin are centered at 283 nm and 341 nm, due to n→p transitions in carbonyl groups of carboxylate or ester moieties [58], as can be seen in Figure 7B for the representative PEC/CMC/OA film (SI9B for all spectra). The spectrum of the PEC/CMC/OA/CaP/AgZ film (spectrum at T0 in Figure 7B) is identical to that of PEC/CMC/OA. The typical LSPR band of silver nanoparticles, centered at 390–400 nm [22], is not present, and it did not form during 48 h of exposition to environmental light (T1 h–T48 h spectra, Figure 7B).

### 2.3. Functional Properties of PEC/CMC Film-Forming Solutions Used as Coatings

In the previous sections it has been demonstrated that the PEC/CMC/OA/CaP and PEC/CMC/OA/CaP/AgZ films have brilliant antimold activity and excellent O_2_ and CO_2_ barrier ability. On the other hand, their permeance to water vapor is high. While the use of such materials as self-standing films can be imagined to increase the shelf life of fresh bread for short periods (days range), it seems less feasible for long-term packaging, e.g., in the case of sliced bread. Considering its matrix of polysaccharides and cellulose, which are biodegradable, the possibility of using our films as a coupled component for paper packaging was investigated from the perspective of increasing the food preservation capacity of this popular material while maintaining its environment-friendly nature. Polysaccharide-based film-forming solutions have been found suitable as coaters for paper [6,7]. Rheological properties are the crucial parameters to choose the correct technique of coating. By investigating the relationship between apparent viscosity and shear rate, we found that for all the PEC/CMC-based films, the apparent viscosity of their film-forming solutions decreases as the shear rate increases, with a shear-thinning behavior (SI10, A). Spraying, brushing, and roller methods require low viscosity values (<0.5 Pa s) at high shear rates (>1000 s^−1^) [59]. These would not be the methods of choice for most of the PEC/CMC films, as only PEC/CMC/OA/CaCl_2_ and PEC/CMC/OA/CaA/Z fit this parameter. Dip-coating necessitates viscosity values > 10 Pa s when low shear rates are applied (10 s^−1^) [59]. All the PEC/CMC films are suitable for this method. The viscous properties of the PEC/CMC film-forming solutions were measured at different shear rates, from 0 s^−1^ to 200 s^−1^ and then back from 200 s^−1^ to 0 s^−1^ at 25(1) °C with a rotational rheometer, and the experimental data were fitted with the Ostwald de Waele model (SI10, B). The results for the model parameters are shown in Table 5. An R^2^ value near 1 was found in all cases, indicating that the Ostwald de Waele model could be confidently used to describe the rheological properties of all the PEC/CMC film-forming solutions. The flow behavior index (*n*) correlates with the concentration of crosslinker ions, decreasing with the addition of Ca^2+^ ions and implying an increase in pseudoplasticity [60]. Furthermore, while neat PEC films without Ca^2+^ have *n* = 0.940(3), the addition of CMC decreases *n* to 0.774(4) in PEC/CMC/OA. The K value corresponds to the consistency of the fluids, with higher K values meaning higher viscosity [60]. Also, in this case (Table 5), the parameter strongly depends on the presence of crosslinker calcium ions, increasing by an order of magnitude from the PEC/CMC/OA film-forming solution, K = 2.01(7), to K = 20(2) in the case of PEC/CMC/OA/CaCl_2_. On changing calcium salts to CaA and CaP and on adding zeolites, the K value further increases, an effect that is connected to the increase in pH in the film-forming solutions (Table 2) that in turn increases the efficiency of the crosslinking between PEC carboxylates with the “egg-box” mechanism, as we have already observed for the barrier effect towards gases.

By calculating the surface area of the hysteresis loop in the shear stress vs. shear rate profiles (SI10, Figure S2B), the thixotropic properties of the film-forming solutions were also determined and added to Table 5. Thixotropy is the property of certain gels of becoming thinner when a constant force is applied, with the viscosity fully recovering the initial state in an appropriate period of time after reduction of the applied force [61,62]. It influences the way in which paints level out, and, when high, it ensures a sufficient and consistent wet layer thickness for coating applications [61,62]. Table 5 shows that thixotropy increases with the addition of crosslinking Ca^2+^ ions, stepping from 566(45) Pa/s for PEC/CMC/OA to 3780(1221)Pa/s for PEC/CMC/OA/CaCl_2_. A further thixotropy increase is observed with the Ca^2+^ salts having a weak base as counter anion (CaA and CaP). As observed for the fluid consistency, this trend is connected to the increased interactions between the polymer chains of pectin due to Ca^2+^ coordination [60]. All the collected data point towards the possibility of using the film-forming solutions of our PEC/CMC/OA/CaP and PEC/CMC/OA/CaP/AgZ as coatings with dip-coating methods.

## 3. Materials and Methods

### 3.1. Materials

Pectin from citrus peel (PEC, galacturonic acid ≥ 74.0% dried basis, methoxy group ≥ 6.7%), carboxymethylcellulose (CMC, average M_w_ ~250,000, degree of substitution 0.7), oleic acid (OA, cis, purity ≥ 90%), zeolites (Z, molecular sieves 4A, powder, ~325 mesh particle size), calcium acetate monohydrate (CaA, CAS 114460-21-8, purity ≥ 99%), and calcium propionate (CaP, CAS 4075-81-4, purity ≥ 97%) were all purchased from Sigma-Aldrich, Milan, Italy. Silver nitrate was purchased from Fluka (Milan, Italy), and calcium chloride was purchased from Merck (Milan, Italy). Commercially available low-density polyethylene (LDPE) films were obtained from Conad (Pavia, Italy) bread packaging, Commercially available MaterBi (MB) was purchased in store.

### 3.2. Methods

#### 3.2.1. Experimental Preparations

Silver-loaded zeolites (AgZ). A total of 0.140 g (0.824 mmol) of AgNO_3_ were dissolved in 100 mL of bi-distilled water at room temperature (RT), and 1.8 g of Z were added to the mixture. The blend was stirred in the dark for 16 h at RT to allow Ag^+^ loading by ion exchange. The AgZ was then filtered and washed with 3 × 15 mL bi-distilled water and dried overnight at 80 °C. They contained 4.9% *w*/*w* Ag with respect to Z mass.

Film preparation. Two solutions were prepared: (i) 3.0 g PEC was dissolved into 50 mL of water at 80 °C under magnetic stirring; (ii) 0.90 g CMC was dissolved in 25 mL of water at 70 °C and kept under magnetic stirring until complete dissolution. A total of 0.30 g of OA was added to the solution of PEC. The two solutions were mixed at 75 °C under magnetic stirring. The following additives were then added:

(a) Ca^2+^ salts: Calcium chloride (CaCl_2_), calcium acetate monohydrate (CaA), or calcium propionate (CaP) were used as crosslinkers. A total of 1.9 mmol was dissolved in 25 mL of water and added to the PEC/CMC solution under vigorous stirring at 75 °C. After 30 min of stirring, the solution was allowed to cool at RT.
(b) AgZ or plain Z in a 10% *w*/*w* proportion with respect to PEC was suspended in 1 mL of water and added at RT.

Precursor solutions for film formation were also prepared with Ca^2+^ salts but without the addition of Z or AgZ. To obtain polymeric films with a precise and homogeneous thickness, we used a tape casting method with a “doctor blade” manual instrument built in our laboratories. The blade was regulated at a 1200 µm height from the base. With the described quantities, 2 sheets of 12 × 20 cm^2^ were prepared. After tape casting, the films were kept in an oven at 40 °C for 18 h. The average final thickness of films was 50(±5) µm.

#### 3.2.2. Characterization of PEC/CMC Films

Thickness. The thickness of the films was measured by using a micrometer Thickness Gauge FD-100 with a resolution of 1 µm (Hans Schimdt & Co. GmbH; D-84478, Waldkraiburg, Germany), averaging five measurements on different points of the same film.

Mechanical properties. The mechanical behavior of the films was evaluated by uniaxial tensile test under ambient conditions (T = 25 °C, RH = 50%) using the TA.HD+ Texture Analyzer testing machine (Stable Micro Systems, Godalming, UK), equipped with a 5 kg load cell. From six to nine specimens (1 × 3 cm^2^) were cut for each film and then clamped on an A/TG tensile grip probe. An initial distance of 1 cm was set between the grips. The upper grip was raised at a constant speed of 0.5 mm/s up to a distance of 10 mm. Young’s modulus (YM) (GPa), tensile strength (TS) (MPa), and elongation at break (E%) (%) were determined from the stress–strain curves.

Barrier properties to oxygen and carbon dioxide. The permeance of the films to oxygen and carbon dioxide ($P_{O_{2}}$ − $P_{CO_{2}}$) was assessed by a manometric method using a GTP-c permeameter (Brugger Feinmachanik GmbH, Munich, Germany) at 25 °C under 0% relative humidity conditions. The system was outgassed under a primary vacuum before testing. At time zero, one side of the film was flushed with the gas (flow 27 cm^3^·min^−1^), and the increase in pressure on the other side was recorded over time. The partial pressure differential was 1000 hPa. The experiments were performed in quadruplicate. The permeance (expressed in mol·m^−2^s^−1^Pa^−1^) was determined from the steady state according to Equation (1), written as follows:

(1) $$P=\frac{∆n}{A\cdot ∆t\cdot ∆p}\;$$

where P is permeance, ∆n is the average molar quantity associated with the gas transfer (mol), A is the surface area of the film (m^2^), ∆t is the time (s), and ∆p is the pressure difference between the two sides of the film (Pa).

Barrier properties to water vapor. The permeance of the films to water vapor ($P_{H_{2}O}$) was determined according to ASTM E96 [63]. This gravimetric method was conducted at 25 °C under two different relative humidity conditions: semi-dry (0–50% RH) and humid (50–100% RH). For this purpose, permeability cells (3.4 cm inner diameter) were used, with the film held between two silicon O-rings at the top and placed in a climatic chamber at 25 °C and 50% RH. To obtain the semi-dry condition, previously dried silica gel was put inside the permeability cell to reach a relative humidity close to 0%. For the humid condition, distilled water was placed in the cells to maintain the relative humidity close to 100%. The cell weight was measured once a day using an analytical balance with an accuracy of 0.1 mg (Sartorius Analytical balance; Sartorius Lab Instruments GmbH & Co. KG—Goettingen, Germany). The permeance to water vapor was determined from the steady state of the transfer according to Equation (1). The analyses were carried out in quadruplicate.

Interaction with light. UV–vis absorption and transmission spectra were recorded on a Varian Cary 6000 spectrophotometer with a dedicated sample holder for films. Transmittance and absorption spectra were recorded between 200 nm and 1100 nm. The evaluation of stability under environmental light was conducted by the exposure of the films to the ambient light of the laboratory (sun and neon), and the absorption spectra were collected after 1, 6, 24, and 48 h. Transparency of the films was given as the percentage of transmittance at 550 nm, and UV screening ability by the percentage at 280 nm. The values obtained were divided by the exact thickness of the measured sample and multiplied by 50 µm for normalization.

Determination of the CaP transfer to bread. Four 5.0 g samples of commercial bread (slices of 3 × 4 × 1.6 cm^3^) were kept in contact for 7 days with a 3 × 4 cm^2^ portion of the PEC/CMC/OA/CaP film (100 mg mass, containing 7.8 mg CaP). The bread samples were soaked in 5.0 mL of water each for two hours, then the solutions were filtered, and the CaP content was analyzed according to a published colorimetric method, used for the quantitative determination of CaP contained in bread [55], by treating each solution with 250 μL of NH_4_Fe(SO_4_)_2_·12H_2_O solution in water(0.8 g/10 mL). No sample gave detectable CaP. The reported method has an LOD of 0.4 mg/mL, so it can be stated that the CaP quantity transferred to bread was <2.0 mg.

Determination of silver release to bread. A total of 100 mg portions (3 × 4 cm^2^) of AgZ-containing film were put in contact with a 3 × 4 × 1.6 cm slice of commercially available bread (5.0 g, Pan Bauletto Bianco, Mulino Bianco, Barilla, Italy) and enveloped in aluminum foil. The samples were kept in a glass bowl with RH 100% at RT and analyzed after 1, 3, and 7 days. At the chosen time, the bread was mineralized (1:5 H_2_O_2_-HNO_3_ at 100 °C for 30 min in a total volume of 10 mL), and 1 mL of the obtained solution was diluted to 5 mL with bidistilled water. The Ag content of the sample was analyzed by ICP-OES on a Perkin Elmer Optima 3300 DV instrument (Milano, Italy). 

In the attempt to mimic a pack of sliced bread, larger portions of each film (12 × 20 cm^2^, 2.0 g) were used to envelop six piled-up 3 × 4 × 1.6 cm^3^ slices of commercial bread (5.0 g each, 30.0 g total mass; Pan Bauletto Bianco, Mulino Bianco, Firenze, Italy). The pack was in turn enveloped in aluminum foil and kept in a glass bowl with RH 100% at RT for 1, 3, and 7 days. After these times, all six slices of bread were mineralized together (1:5 H_2_O_2_-HNO_3_ at 100 °C for 30 min, 50 mL total volume), and 5.0 mL of the obtained solution were diluted to 25 mL and the Ag content was analyzed by ICP-OES on a Perkin Elmer Optima 3300 DV instrument.

Morphology characterization and quantification of silver. Morphology characterization was carried out by scanning electron microscopy (SEM). For samples containing Z and AgZ, ~5 mg of each sample were directly attached onto the stub by using carbon tape and then sputtered with carbon and imaged with a Scanning Electron Microscope (SEM) Zeiss EVO MA10 (Carl Zeiss, Oberkochen, Germany). The SEM-EDS analysis of Z and AgZ was obtained with the same instrument, coupled with Xmax microprobe (Oxford, UK). The images were acquired at high voltage (20 kV), in high vacuum, at room temperature, and at different magnifications. The SEM images of the films were achieved with a Tescan Mira XMU-FEG SEM instrument (Brno, Czech Republic) on films (1 × 1 cm^2^) attached to the stub with carbon tape and then sputtered with carbon (thickness ≤ 10 nm). The films were also analyzed by SEM-EDS as previously described.

Antimicrobial activity test. The antimicrobial activity of the films containing either CaP or CaP and AgZ was evaluated against three fungal strains: *Aspergillus niger* ATCC 16404, *Penicillium janthinellum* ATCC 20312, and *Penicillium* spp. wild type. Fungal suspensions were filtered with an initial inoculum of approximately 1–2 × 10^5^ CFU/mL (colony forming unit/mL) on filter membranes of cellulose acetate with a porosity of 0.22 µm. The membranes were then deposited on Petri plates containing a culture medium/agar suitable for the growth of microorganisms selected. The filter membranes were covered with films containing CaP or CaP and AgZ films for 96 h. Similarly, plates were prepared containing membrane filters covered with a control film, prepared with CaCl_2_ as the crosslinker and containing no AgZ or CaP. After the contact time, the filter membranes were recovered and washed by suspending them in sterile water with a standardized method [64]. After washing, dilutions of the microbial suspensions were made to determine the microbial content with subsequent sowing in a plate and calculation of the microbicidal effect (ME). This was calculated for each test organism and contact time according to the following equation:

ME = log(N_c_/N_d_)

where Nc is the number of CFU of the microbial suspension after contact with the control film, and Nd is the number of CFU of the microbial suspension after contact with the CaP or CaP and AgZ film.

Fourier-transform infrared spectroscopy (FTIR). FTIR analysis was carried out with a Bruker IFS 28 (Milano, Italy) equipped with a DTGS detector and working in a spectral range of 4000–600 cm^−1^, with a resolution of 2 cm^−1^. A total of 32 scans were collected to obtain a high signal-to-noise ratio.

Shelf life evaluation of bread slices. The bread used for this experiment was supplied by Millbo s.r.l. (Trecate, Italy) and was prepared without any type of preservatives.

- A total of 30 slices of bread (3 × 3 cm^2^ each) were packed in common LDPE film closed with a heat sealer.
- A total of 30 slices of bread (3 × 3 cm^2^ each) were packed and sealed in a CaP film and then further packed in an external LDPE bag.
- A total of 30 slices of bread (3 × 3 cm^2^) were packed in a CaP + AgZ film and then further packed in an external LDPE bag. All the bags were maintained in the dark at a controlled temperature (25 °C), and visual evaluation of the presence of molds was conducted twice a day. The shelf life experiment was repeated three times.

Thermal analysis. The evolution of the thermal events of the films was studied by Differential Scanning Calorimetry (DSC) using a Q20 calorimeter (T.A. Instruments, New Castle, DE, USA). Film samples were weighed (3–5 mg) and sealed into aluminum pans (T-zero, T.A. Instruments, New Castle, DE, USA) before being subjected to a double heating–cooling cycle at 10 °C·min^−1^ under N_2_ atmosphere (flow rate = 25 mL·min^−1^), from 0 to 300 °C.

### 3.3. Characterization of the Film-Forming Solutions

The pH of the film-forming solutions was measured using a pH meter (Instruments pH-meter (pH 50 model) with a Thermo Scientific Orion 91022 BNWP (Monza, Italy) combined glass electrode). Electrode calibration was carried out before measurements, with solutions buffered at pH = 4, pH = 7, and pH = 10. The samples were equilibrated to ambient conditions (T ≈ 25 °C) before analysis. The analysis was performed in triplicate.

Rheological properties. A rotational rheometer (HAAKE^TM^ RotoVisco^TM^ 1; Thermo Fisher Scientific, Boston, MA, USA) was used to determine the rheological properties of the solutions at 25 ±1 °C. For the analysis, a Z34DIN sensor with a rotor No. 222-1499 (radius = 17.000 mm, length 51 mm, clearance bottom 7.2 mm) and a cup No. 222-1498 (radius = 18.44 mm), which makes a gap of 1.44 mm between the rotor and the cup. The rheological properties were measured at different shear rates from 0 s^−1^ to 200 s^−1^. The measurements were performed in triplicate. The experimental data of all the solutions’ flow curves were analyzed using the Ostwald de Waele model fitting, written as follows:

(2) $$\tau ={\kappa \dot{\gamma \;}}^{n}$$

where τ is the shear stress (Pa), $\dot{\gamma \;}$ is the shear rate (s^−1^), κ is the consintency index (Pa·s^n^), and n is the flow behavior index (dimensionless).

The apparent viscosity of the film-forming solutions was extrapolated using the κ and n  constants that were previously calculated to predict the apparent viscosity of the solutions at 1000 s^−1^. The values of apparent viscosity at 10 s^−1^ and 1000 s^−1^ were chosen for the comparison of the different solutions.

### 3.4. Statistical Analysis

Statistical analysis of the data was performed using IBM (Armonk, NY, USA) SPSS statistics 20 software. The data were ranked, and statistical differences were evaluated using a one-way analysis of variance (ANOVA) and Tukey’s multiple comparison tests. In all cases, a *p*-value < 0.05 was considered significant.

## 4. Conclusions

PEC and CMC are biodegradable polymers obtained from renewable sources. In this paper it has been shown that new self-standing films based on a mixture of the two polymers can be obtained with enhanced preservative features using OA as a non-hydrophilic plasticizer, CaP as the crosslinker, and AgZ as an Ag^+^ releasing additive. The combination of these components leads to a satisfactory mechanical behavior of the films and to excellent gas barrier properties for O_2_ and CO_2_. Comparison with the gas barrier properties of the market-standard films LDPE (currently used for bread packaging) and MB (a commonly used material for biodegradable sachets) may have some limitations—due to the presumably different preparation techniques of the two commercial films—but impressively lower (3–4 orders of magnitude) CO_2_ and O_2_ permeance values are found anyway. It has also been found that due to the hydrophilic nature of PEC and CMC, the water vapor barrier exerted by the films remains instead low, i.e., far from that of LDPE and comparable to that of MB. From the preservation point of view, the films prepared with CaP as the crosslinker exert a drastic anti-mold effect, both on mold lines inoculated on a membrane and on real bread samples. Significantly, such an effect is obtained by avoiding the addition of the CaP preservative in the bread and moving it to the packaging, this allowing only trace quantities (<0.04% *w*/*w*) to be released during contact. The preservative properties of this active packaging, PEC/CMC/OA/CaP, are further improved by adding a source of the antimicrobial Ag^+^ ions that are released in very small traces from the films. This is an innovation: while Ag^+^ release from active packaging to food is commonly obtained using AgNP, in this research nanoparticles have been intentionally avoided, as they are generally not allowed as food contact materials by regulatory agencies. AgZ were instead embedded in the films, i.e., micrometric zeolite powders were used, loaded with Ag^+^ cations. Moreover, it has been experimentally verified that the Ag^+^ released from PEC/CMC/OA/CaP/AgZ to bread is lower than 0.05 mg/Kg of bread, which is the quantity allowed by EU regulation [39]. Finally, it has been found that the PEC/CMC/OA/CaP and PEC/CMC/OA/CaP/AgZ films are excellent UV filters while they are transparent in the visible, allowing visual examination of the enveloped food.

While the two PEC/CMC/OA/CaP and PEC/CMC/OA/CaP/AgZ films are not competitive with the currently used LDPE packaging for bread, if a very long shelf life is needed (e.g., weeks, typically for sliced bread), their renewable feedstock origin, their biodegradable nature, their composition, and features well within the limits imposed by regulatory agencies make these films good candidates for a new generation of packaging material, with the elimination of high-dosage preservatives inside bread. As a matter of fact, they prevent mold formation, prolong the shelf life of bread, and comply with the consumer demands for clean-label foods (i.e., with no added preservatives) and more sustainable packaging.

## 5. Patents

An Italian patent was granted based on the work described in this paper: Pallavicini, P.; Doveri, L.R. *Film per la conservazione di alimenti*, 102021000032831 (granted 2024)—an international patent was filed (PCT/IB2022/062808, currently under examination).

## Figures and Tables

**Table 1:** Mechanical properties of PEC/CMC films, MB, and LDPE.

Films	Thickness (µm)	Young’s Modulus (GPa)	Tensile Strength (MPa)	Elongation at Break (%)
MB	12.6(±0.5) ^a^	0.80(±0.03) ^c,d^	26(±1) ^b,c^	198(±28) ^c^
LDPE	51(±1) ^c,d^	0.26(±0.03) ^a^	18(±2) ^a^	1206(±121) ^d^
PEC/CMC/OA	47(±2) ^b,c,d^	1.35(±0.09) ^e^	35(±5) ^b,c,d^	2.6(±7) ^a^
PEC/CMC/OA/CaCl_2_	54(±7) ^d^	0.56(±0.05) ^b^	51(±7) ^b,c,d^	7(±1) ^b^
PEC/CMC/OA/CaA	65(±7) ^e^	0.75(±0.07) ^b,c,d^	59(±15) ^c,d^	8(±1) ^b^
PEC/CMC/OA/CaA/Z	64(±7) ^e^	0.69(±0.05) ^b,c,d^	55(±16) ^c,d^	8(±1) ^b^
PEC/CMC/OA/CaA/AgZ	47(±5) ^b,c,d^	0.60(±0.01) ^b,c^	54(±11) ^b,c,d^	8(±1) ^b^
PEC/CMC/OA/CaP	43(±4) ^b,c^	0.85(±0.06) ^d^	63(±15) ^d^	8(±2) ^b^
PEC/CMC/OA/CaP/Z	40(±5) ^b^	0.70(±0.01) ^b,c,d^	50(±16) ^b,c,d^	9(±3) ^b^
PEC/CMC/OA/CaP/AgZ	50(±4) ^c,d^	0.60(±0.01) ^b,c^	54(±11) ^b,c,d^	8(±1) ^b^

Each value is the average of the measurements taken on five samples. Values are reported as mean (standard deviation). Different Roman letters correspond to the classification groups within the same column for the ANOVA Tukey’s test (*p* < 0.05).

**Table 2:** pH of the film-forming solutions.

Film-Forming Solutions	pH
PEC/CMC/OA	3.89(±4) ^b^
PEC/CMC/OA/CaCl_2_	3.50(±1) ^a^
PEC/CMC/OA/CaA	4.40(±8) ^c^
PEC/CMC/OA/CaA Z	5.00(±2) ^d^
PEC/CMC/OA/CaP	4.80(±3) ^d^
PEC/CMC/OA/CaP Z	5.71(±7) ^e^

Each value is the average of the measurements taken on three samples. Values are reported as mean (standard deviation). Different alphabetical letters in each column indicate significant (*p* < 0.05) differences between means using Tukey’s test.

**Table 3:** Antimold activity of PEC/CMC/OA/CaP and PEC/CMC/OA/CaP/AgZ against different fungal strains after 96 h of contact; each value is the average of three measurement repetitions, with standard deviation in parentheses.

Fungal Strain	PEC/CMC/OA/CaP	PEC/CMC/OA/CaP/AgZ
	ME	ME
*Aspergillus niger*	3.36 (±0.34)	3.64 (0.23)
*Penicillium janthinellum*	2.7 (0.42)	4.71 (0.44)
*Penicillium* supp. Wild type	2.1 (0.56)	3.94 (0.62)

**Table 4:** Release of silver from PEC/CMC/OA/CaP/AgZ composite film.

Days of Contact	B: Small Samples, 5 g of Bread	C: Piled Samples, 30 g of Bread
	mg Ag/kg Bread	mg Ag/kg Bread
1	0.05(±0.01)	0.043(±0.004)
3	0.06(±0.02)	0.030(±0.005)
7	0.05(±0.01)	0.03(±0.01)

Each value is the average of the measurements taken on three samples. Values are reported as mean ± standard deviation.

**Table 5:** Fitting parameters of the Ostwald de Waele model for pectin film-forming solutions and thixotropy values.

*Film-Forming Solutions*	*Ostwald de Waele Model Fitting Parameters*			Thixotropy (ΔA Pa/s)
	K	N	R^2^	
PEC/CMC/OA	2.01(±7) ^a^	0.744(±4) ^d^	0.99(±1)	566(±45) ^a^
PEC/CMC/OA/CaCl_2_	20(±2) ^b^	0.45(±2) ^c^	0.9991(±3)	3780(±1221) ^b^
PEC/CMC/OA/CaA	31(±3) ^c^	0.44(±1) ^b,c^	0.9988(±5)	3812(±691) ^b^
PEC/CMC/OA/CaA/Z	57(±3) ^e^	0.308(±7) ^a^	0.996(±2)	5878(±488) ^c^
PEC/CMC/OA/CaP	40(±1) ^d^	0.422(±4) ^b^	0.9989(±2)	4595(±294) ^b,c^
PEC/CMC/OA/CaP/Z	35.1(±6) ^c^	0.413(±2) ^b^	0.9992(±5)	3993(±232) ^b^

Each value is the average of the measurements taken on four samples. Values are reported as mean (standard deviation). Different alphabetical letters in each column indicate significant (*p* < 0.05) differences between means using Tukey’s test.

## Data Availability

Data are contained within the article and Supplementary Materials.

## Supplementary Materials

The following supporting information can be downloaded at https://www.mdpi.com/article/10.3390/molecules30112257/s1: mechanical properties of PEC/CMC/AO/CaCl_2_ films as a function of CMC and Ca^2+^ quantities; “Doctor blade” instrument and film photos; mechanical properties of the films without CMC; barrier properties (O_2_, CO_2_, and water vapor); pH of the film-forming solutions (no CMC added); SEM-EDS analysis; SEM imaging of the films; FT-IR spectra; transmission and absorption spectra; rheological properties of the film-forming solutions.
