# Supplementary material for: Active Pectin/Carboxymethylcellulose Composite Films for Bread Packaging
Source: Molecules. 2025 May 22;30(11):2257. doi: 10.3390/molecules30112257 (PMC12155863; doi:10.3390/molecules30112257)
Supplement: Supplementary file 1 [file molecules-30-02257-s001.zip › molecules-3579703-supplementary.pdf]

Supplementary Information

Active pectin/carboxymethylcellulose composite films for bread packaging

SI1: Preliminary studies on PEC/CMC formulations by varying % CMC and the quantity of $CaCl_2$

films		**Thickness (µm)**	**Tensile Strength(MPa)**	**Elongation at break%**
PEC/CMC 10%/OA	Ca^{2+} 0.75mmol	50(7)	31(27)	8(2)
	Ca^{2+} 1.10mmol	65(5)	28(11)	6(2)
	Ca^{2+} 1.5mmol	70(5)	34(8)	7(3)
PEC/CMC 20%/OA	Ca^{2+} 0.75mmol	60(7)	32(13)	4(1)
	Ca^{2+} 1.10mmol	60(10)	28(7)	6(1)
	Ca^{2+} + 1.5mmol	70(9)	38(18)	7(4)
PEC/CMC 30%/OA	Ca^{2+} 0.75mmol	50(5)	54(14)	9(1)
	Ca^{2+} 1.10mmol	55(9)	54(15)	6(2)
	Ca^{2+} 1.5mmol	50(5)	57(10)	7(4)
	Ca^{2+} 1.9mmol	54(7)	51(7)	7(1)
PEC/CMC 40%/OA	Ca^{2+} 0.75mmol	70(10)	48(16)	8(3)
	Ca^{2+} 1.10mmol	60(9)	44(20)	7(4)
	Ca^{2+} 1.5mmol	80(10)	40(13)	7(2)

Each value is the average of the measurements taken on four samples. Values are reported as mean (standard deviation).

SI2: photos of the “doctor blade” instrument and of the produced films

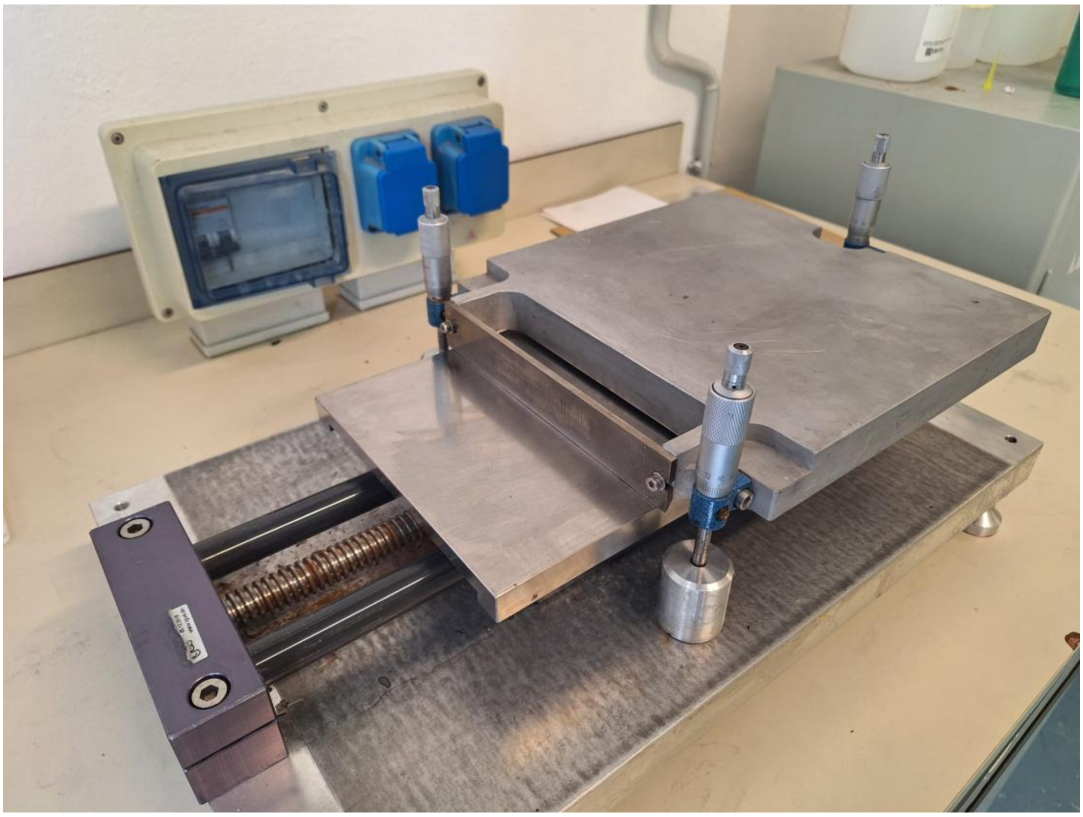

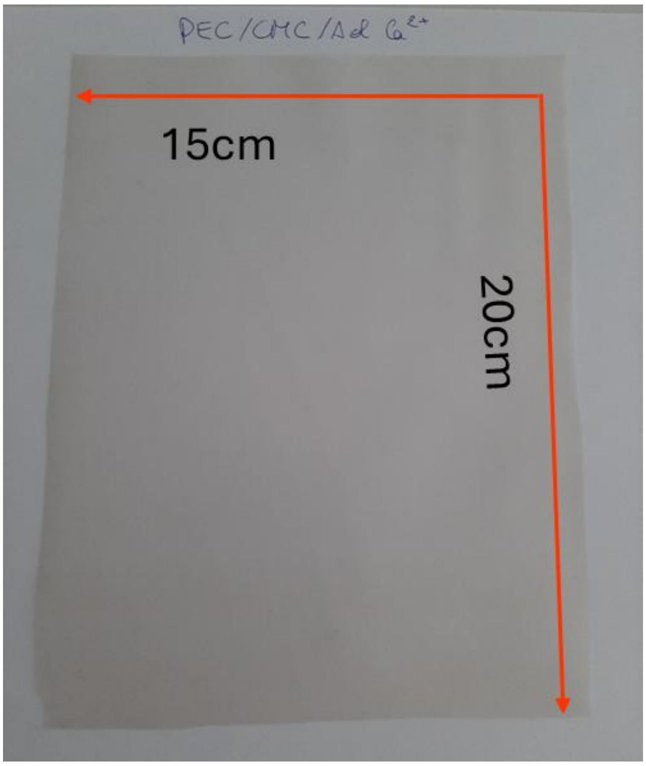

SI3:Mechanical properties of Pectin films (without CMC)

Films	**Thickness (μm)**	**Young's Modulus (GPa)**	**Tensile Strength (MPa)**	**Elongation at break (%)**
MB	12.6(5)^{a}	0.80(3)^{a}	26(1)^{a}	198(28)^{a}
PEC/OA/$CaCl_2$	46(5)^{b}	2.2(2)^{b}	30(7)^{a,b}	1.6(3)^{b}
PEC/OA/CaA	39(3)^{c}	2.9(1)^{d,e}	43(5)^{a,b}	1.5(3)^{b}
PEC/OA/CaA/Z	48(1)^{b}	2.3(5)^{b,c}	27(9)^{a}	1.3(3)^{b}
PEC/OA/CaP	38(1)^{c}	3.4(6)^{e}	46(13)^{b}	1.4(4)^{b}
PEC/OA/CaP/Z	50(2)^{b}	2.7(3)^{b,c}	36(12)^{a,b}	1.6(5)^{b}

Each value is the average of the measurements taken on five samples. Values are reported as mean (standard deviation). Different roman letters correspond to the classification groups within the same column for the ANOVA Tukey's test ($p < 0.05$).

SI4- Barrier properties of PEC/CMC composite films, LDPE and MB

	Oxygen permeance (mol m^{-2} s^{-1} Pa^{-1})	Carbon dioxyde permeance (mol m^{-2} s^{-1} Pa^{-1})	Water vapor permeance (mol m^{-2} s^{-1} Pa^{-1})	
Films	RH=0%	RH=0%	RH=0-50%	RH=50-100%
LDPE	1.90(9)e-11 ^{f}	7.6(6)e-11 ^{c}	1.85(5)e-09 ^{a}	1.39(5)e-09 ^{a}
MB	4.0(7)e-11 ^{e}	1.8(2)e-10 ^{d}	4.0(2)e-08 ^{b}	8.9(6)e-08 ^{b}
PEC/CMC/OA	4.8(1)e-14 ^{d}	5.4(9)e-14 ^{b}	7.2(2)e-08 ^{d,e}	2.68(5)e-07 ^{e,f}
PEC/CMC/OA/$CaCl_2$	1.3(3)e-14 ^{c}	1.7(3)e-14 ^{a}	7.2(2)e-08 ^{c,d,e}	2.3(1)e-07 ^{c,d}
PEC/CMC/OA/CaA	8.4(6)e-15 ^{a,b,c}	1.9(6)e-14 ^{a}	7.3(2)e-08 ^{e}	2.49(9)e-07 ^{e,d}
PEC/CMC/OA/CaA/Z	3.6(7)e-15 ^{a}	1.7(5)e-14 ^{a}	7.71(2)e-08 ^{e}	2.9(1)e-07 ^{f,g}
PEC/CMC/OA/CaA/AgZ	7.5(9)e-15 ^{a,b,c}	1.5(8)e-14 ^{a}	7.4(2)e-09 ^{e}	2.97(2)e-07 ^{g}
PEC/CMC/OA/CaP	6.7(8)e-15 ^{a,b}	1.1(7)e-14 ^{a}	3.53(4)e-08 ^{b}	2.11(6)e-07 ^{c}
PEC/CMC/OA/CaP/Z	1.4(9)e-15 ^{a}	1.4(8)e-14 ^{a}	6.43(2)e-09 ^{c}	2.28(4)e-07 ^{c,d}
PEC/CMC/OA/ CaP/AgZ	9.5(6)e-15 ^{b,c}	1.79(9)e-14 ^{a}	6.5(1)e-08 ^{c,d}	2.81(6)e-07 ^{f,g}

Each value is the average of the measurements taken on four samples. Values are reported as mean (standard deviation).
Different alphabetical letters in each column indicate significant (P < 0.05) differences between mean using Tukey's test.

SI5: Barrier properties of PEC composite film (no CMC added)

Films	Oxygen permeance (mol m^{-2} s^{-1} Pa^{-1})	Water vapor permeance (mol m^{-2} s^{-1} Pa^{-1})	
	RH=0%	*RH=0-50%*	*RH=50-100%*
PEC/OA/$CaCl_2$	2.1(7)e-14 ^{c}	1.05(9)e-07 ^{c}	3.0(1)e-07 ^{f}
PEC/OA/CaA	1.1(1)e-14 ^{d}	9.6(5)e-08 ^{c}	2.6(1)e-07 ^{d}
PEC/OA/CaA/Z	7.3(5)e-15 ^{d}	9.9(7)e-08 ^{c}	2.78(7)e-07 ^{d,e}
PEC/OA/CaP	6(1)e-15 ^{d}	9.3(3)e-08 ^{c}	2.29(7)e-07 ^{c}
PEC/OA/CaP/Z	4(1)e-15 ^{d}	9.9(2)e-08 ^{c}	3.2(2)e-07 ^{f}

Each value is the average of the measurements taken on four samples. Values are reported as mean (standard deviation). Different alphabetical letters in each column indicate significant (P < 0.05) differences between mean using Tukey's test.

SI6: pH of the film-forming solutions (formulations without CMC)

film forming solutions	pH
PEC	3.47(6) ^{b}
PEC/OA/$CaCl_2$	3.19(6) ^{a}
PEC/OA/CaA	4.42(9) ^{c}
PEC/OA/CaA/Z	4.60(4) ^{c}
PEC/OA/CaP	4.6(1) ^{c}
PEC/OA/CaP/Z	5.11(9) ^{c}

Each value is the average of the measurements taken on three samples. Values are reported as mean (standard deviation). Different alphabetical letters in each column indicate significant ($P < 0.05$) differences between mean using Tukey's test

SI7: SEM-EDS analysis

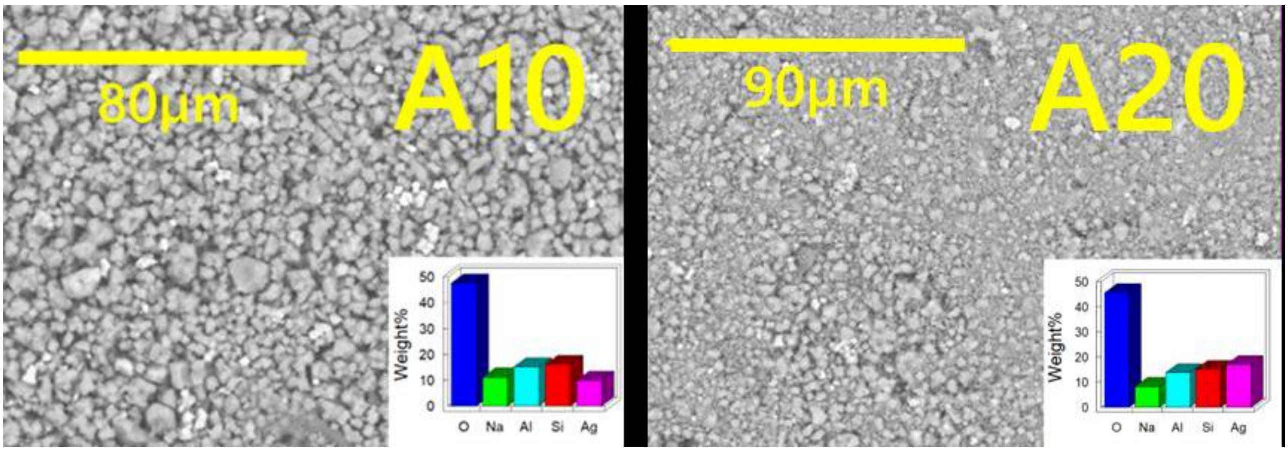

SEM images of Ag^{+}-loaded zeolites. A10: 10% w/w silver. A20: 20% w/w silver. Insets show the respective EDS analysis

SI8: SEM images of the films (same as in Figure 4, main text; larger images)

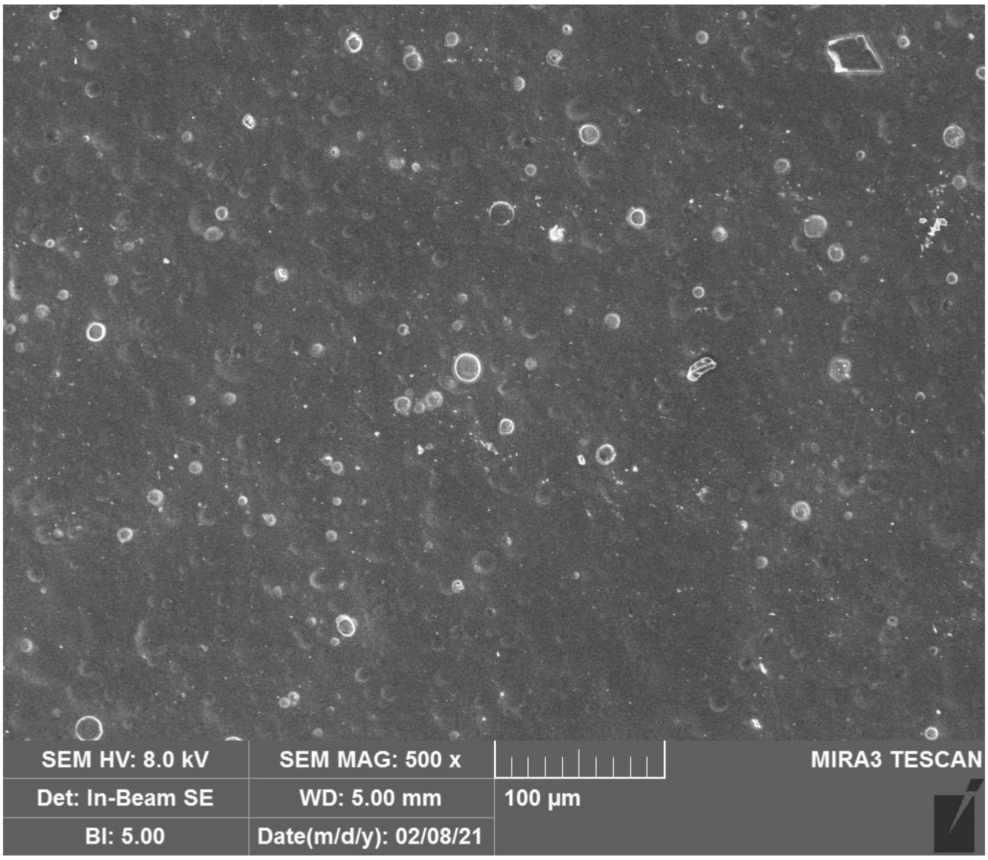

SI8 - A1 - PEC/CMC/OA/$CaCl_2$, surface

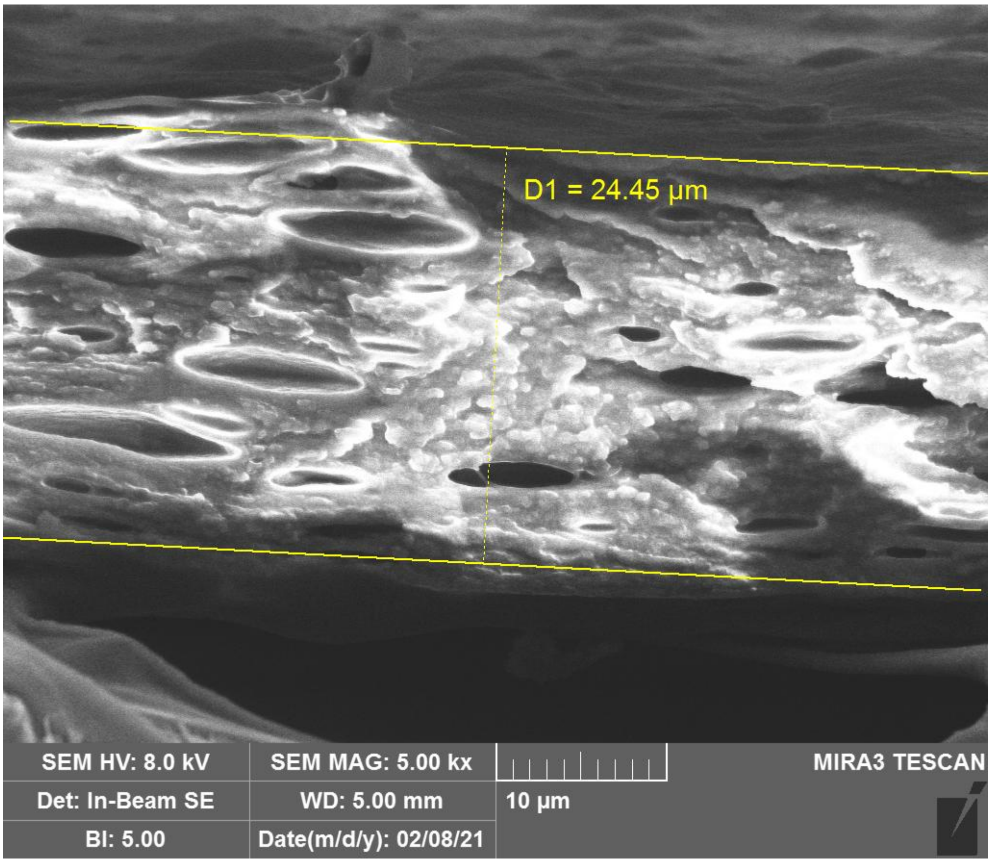

SI8 - A2 - PEC/CMC/OA/$CaCl_2$, section

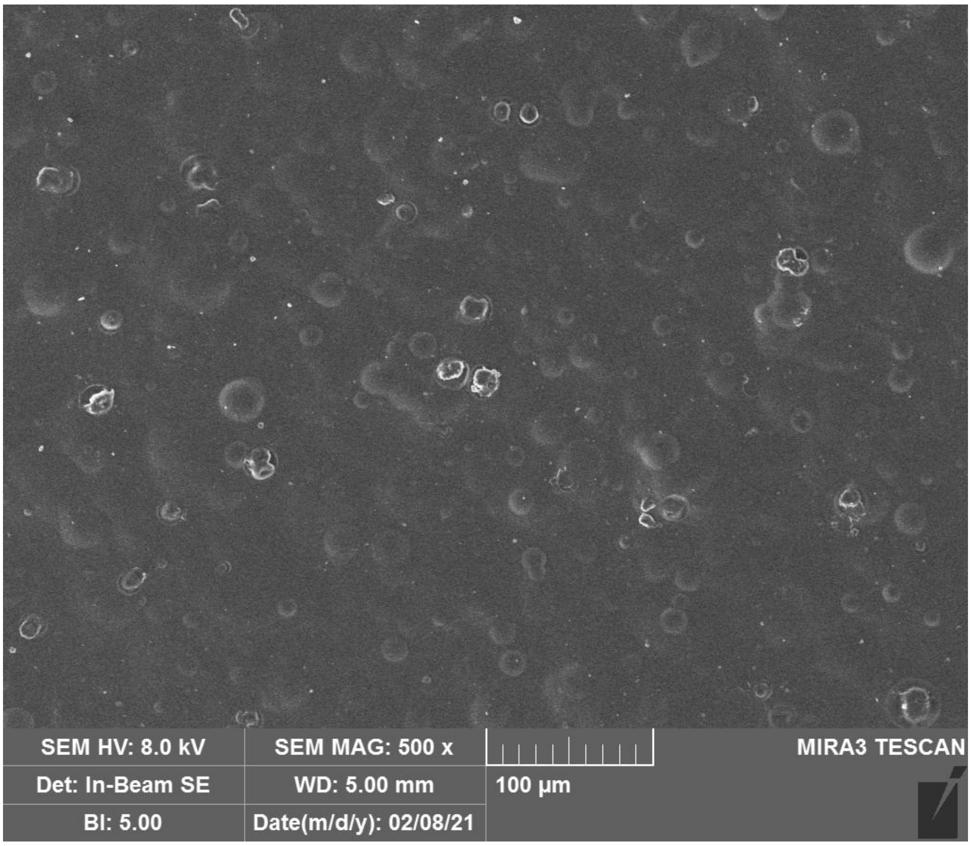

SI8 – B1 - PEC/CMC/OA/CaA surface

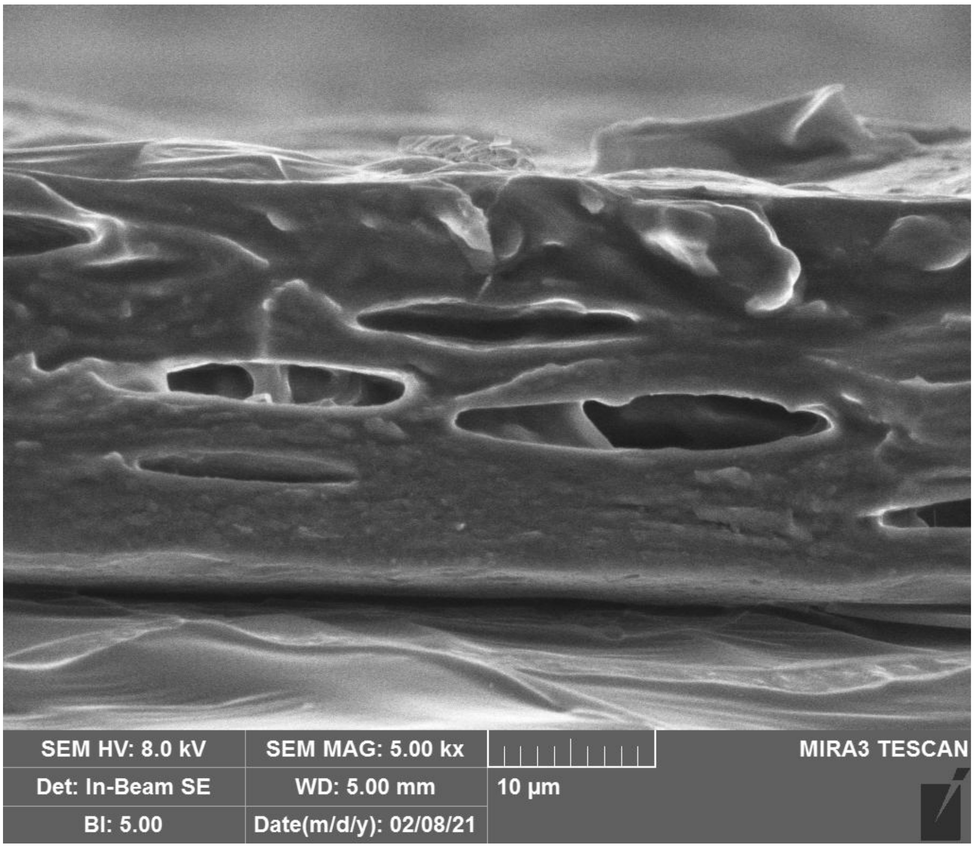

SI8 – B2 - PEC/CMC/OA/CaA section

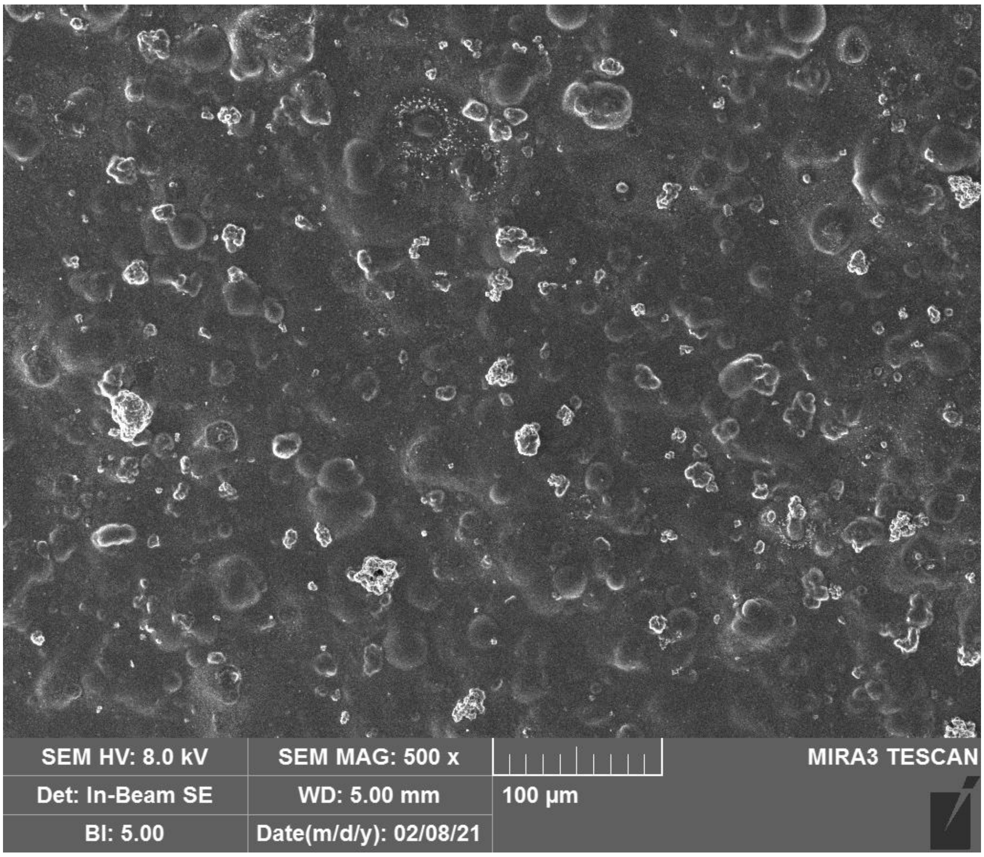

SI8 – C1 - PEC/CMC/OA/CaA/AgZ surface

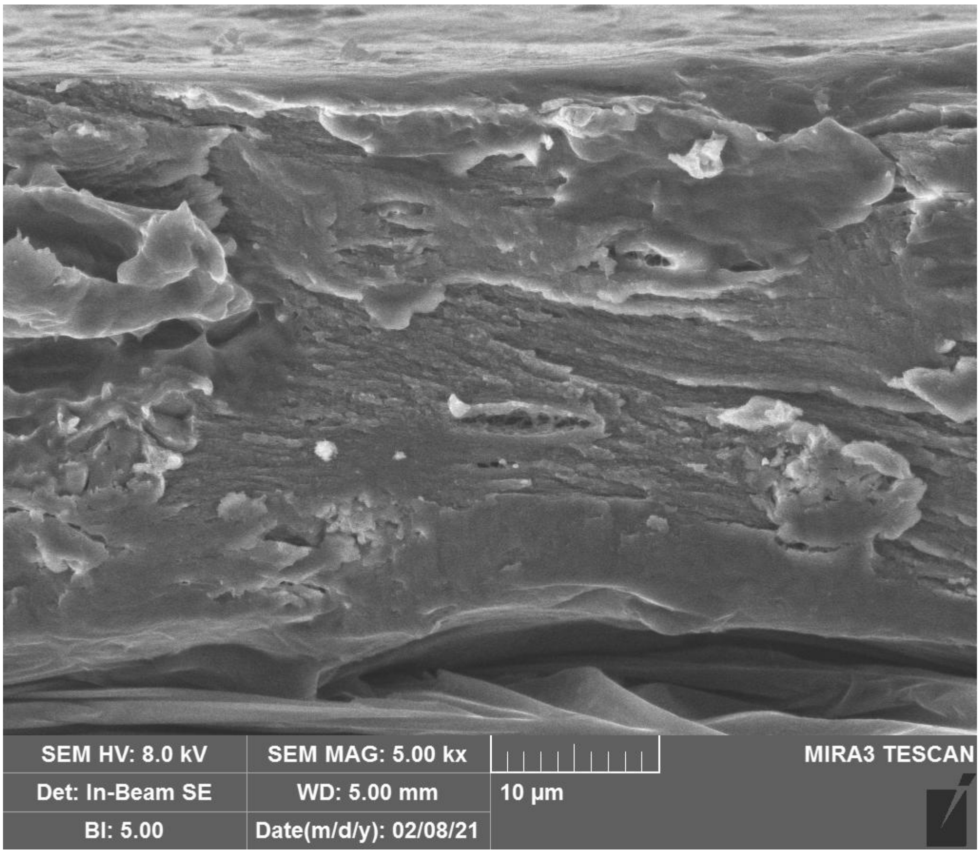

SI8 – C2 - PEC/CMC/OA/CaA/AgZ section

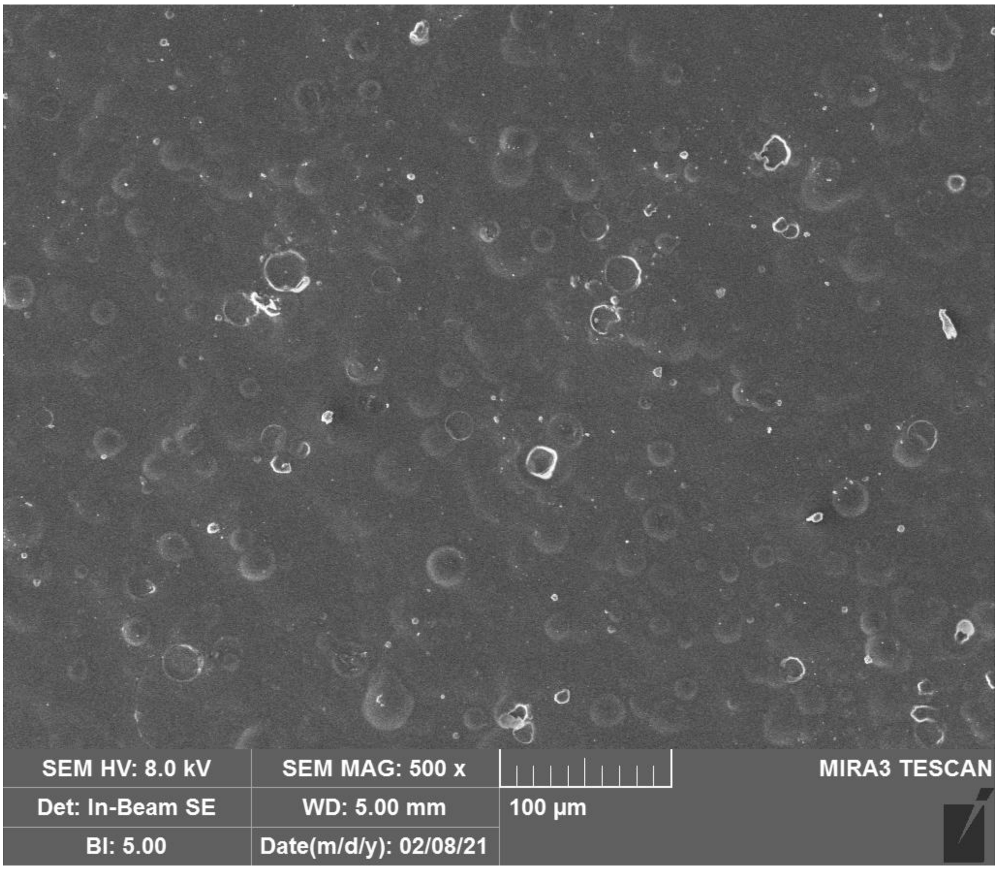

SI8 – D1 - PEC/CMC/OA/CaP surface

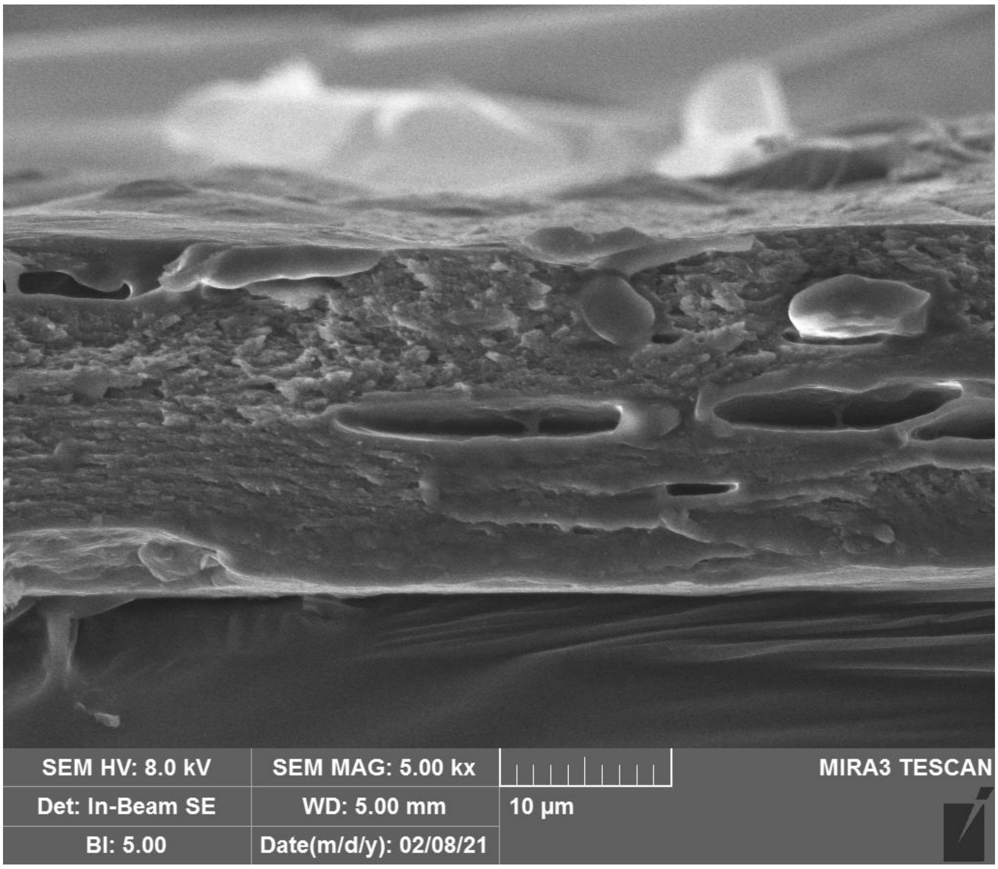

SI8 – D2 - PEC/CMC/OA/CaP section

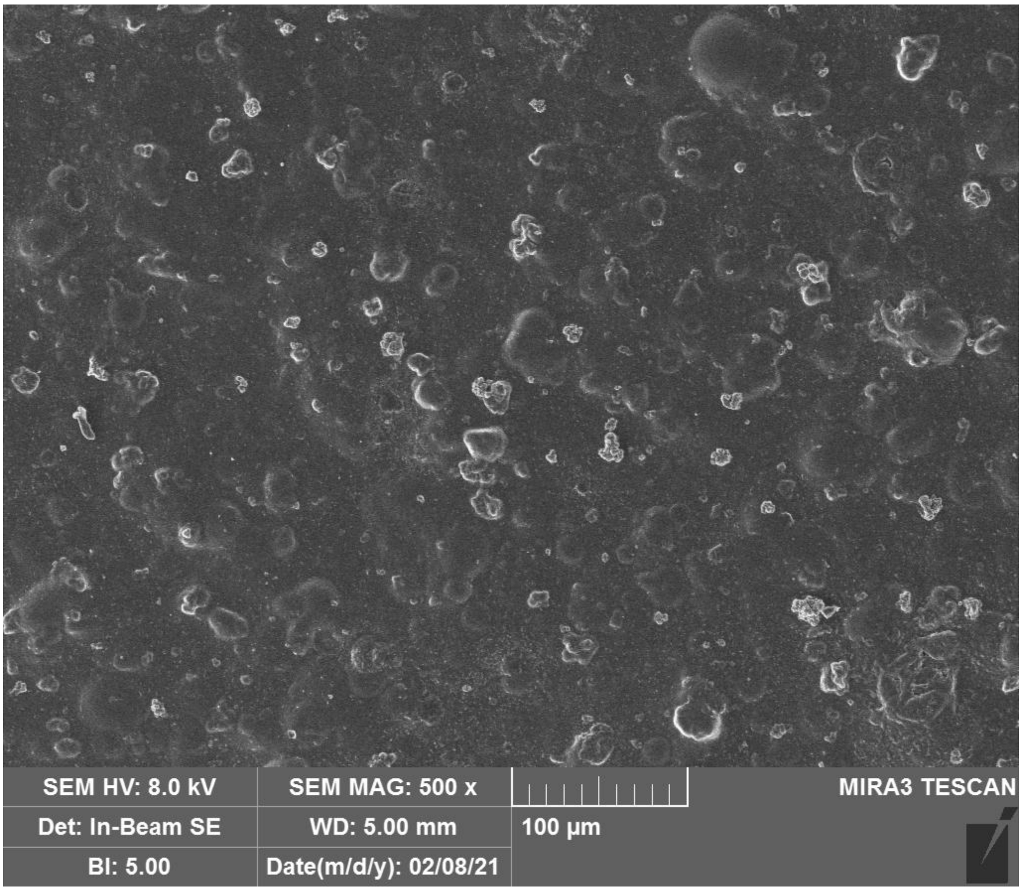

SI8 – E1 - PEC/CMC/OA/CaP/AgZ surface

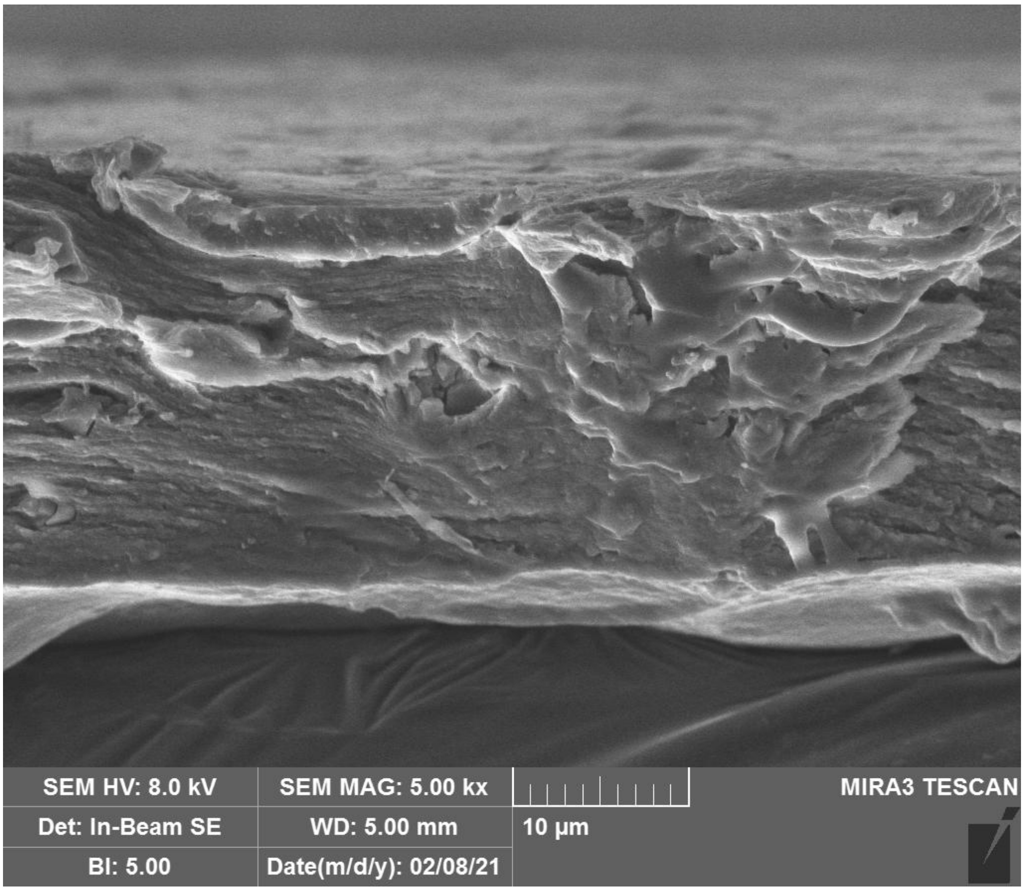

SI8 – E2 - PEC/CMC/OA/CaP/AgZ section

SI9 – Transmission and absorption spectra of the films

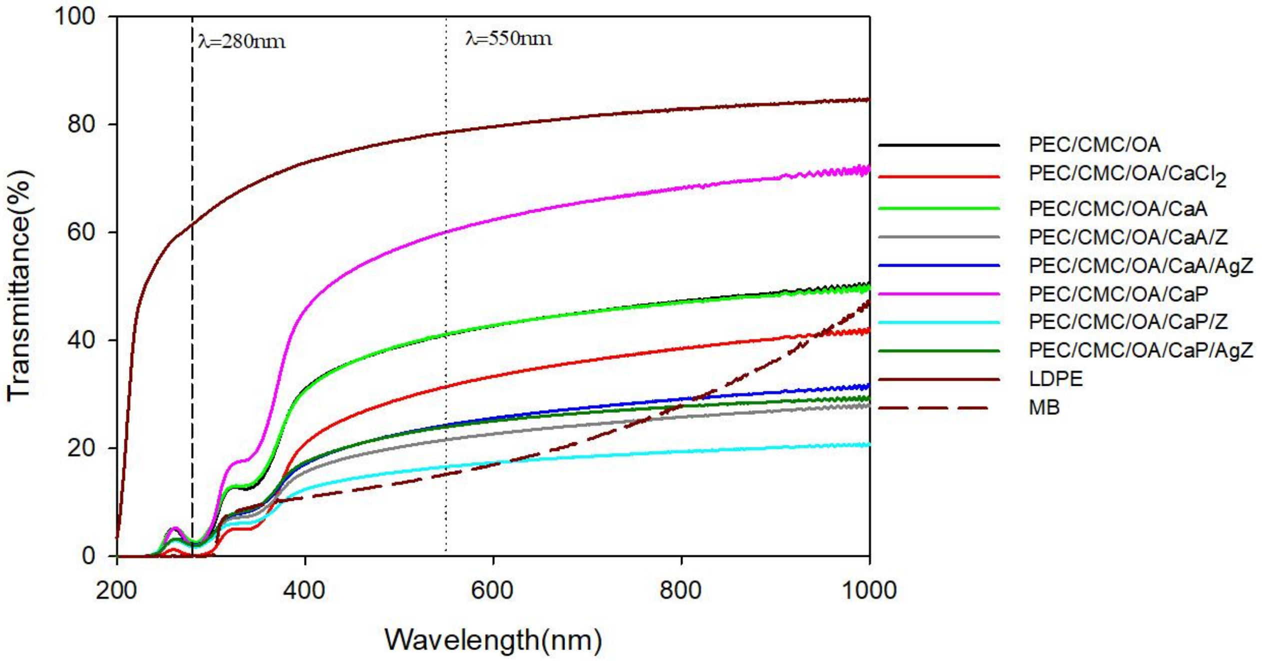

Figure S1A. Transmission spectra of the films

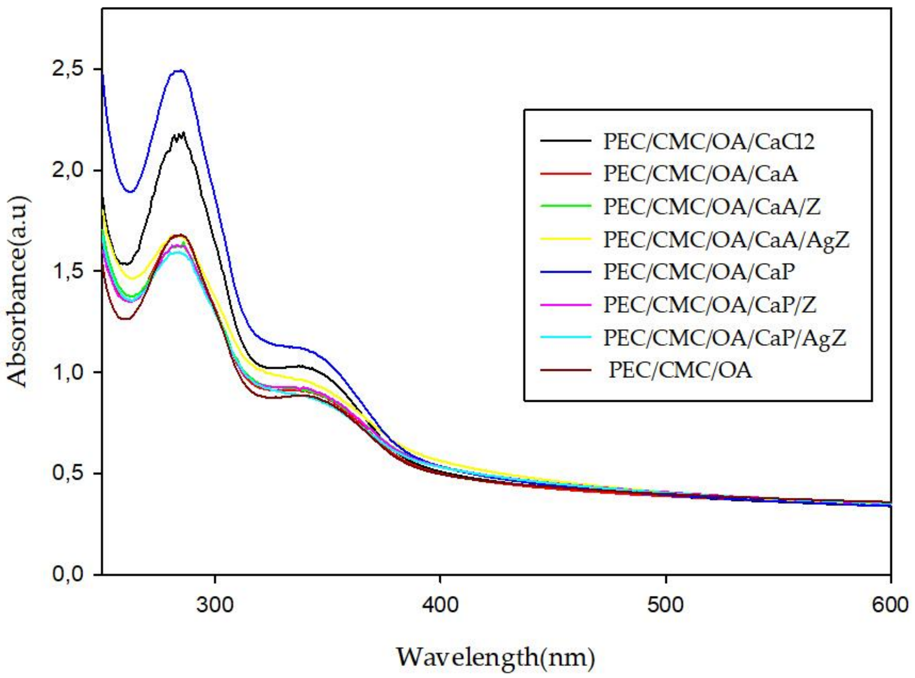

Figure S1B. Absorption spectra of the films

SI10 – Rheological properties of the film-forming solutions

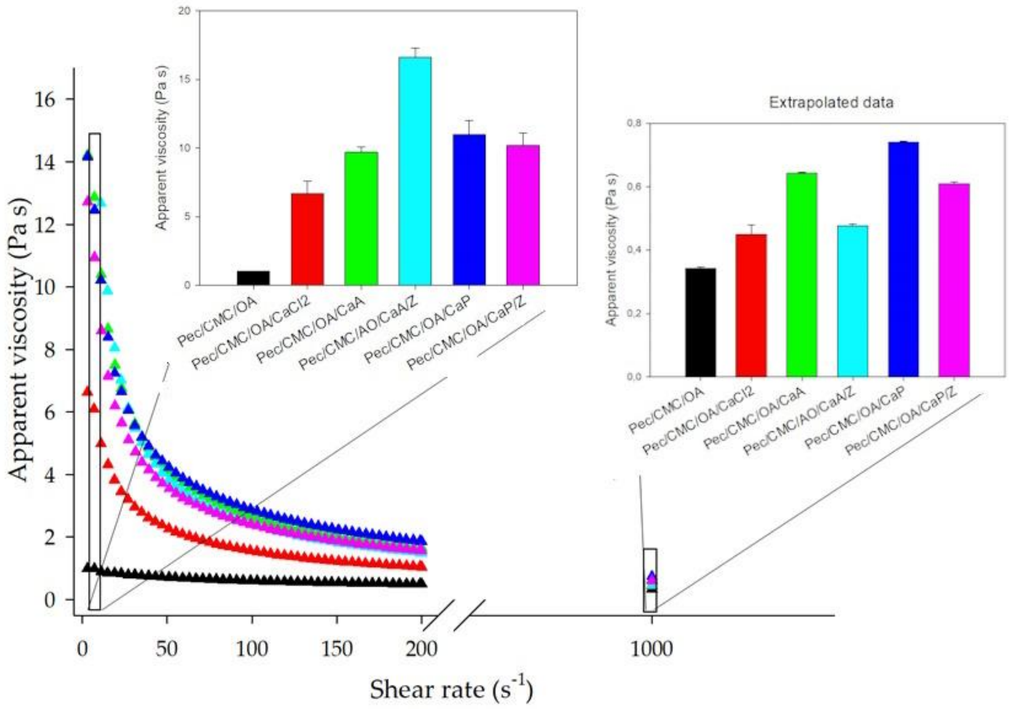

Figure S2A -Apparent viscosity as function of shear rate of all the composite film forming solution, measured at 25±1°. Values of the apparent viscosity at 1000s-1 were calculated by extrapolation of the data. Values are a mean of 3 measures

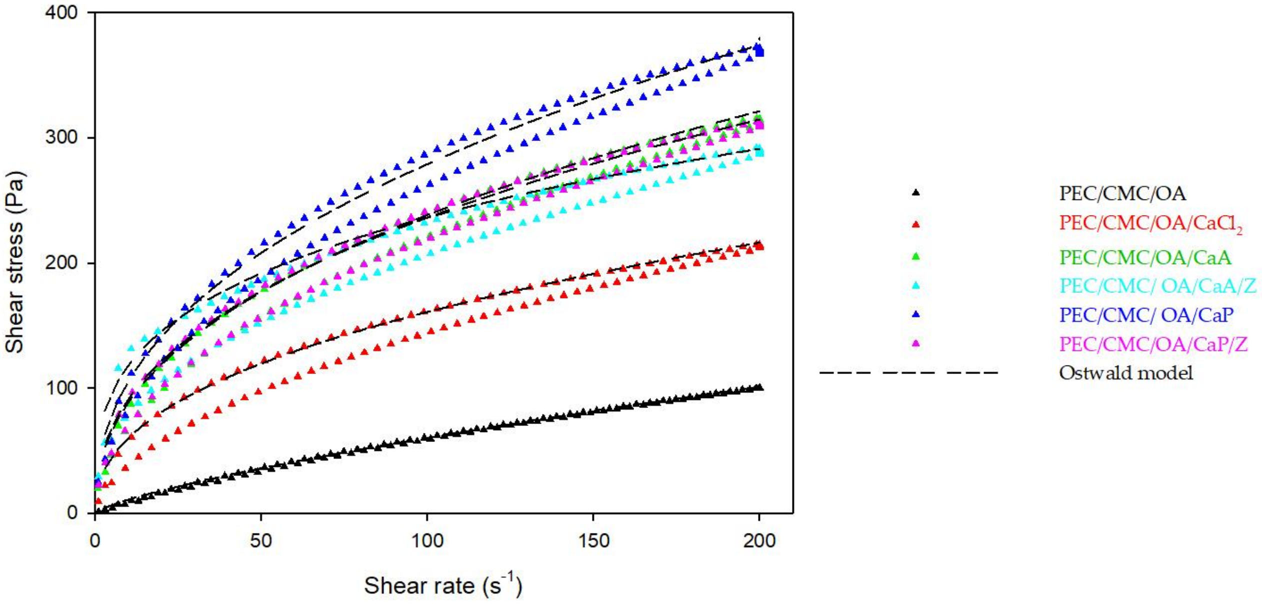

Figure S2B - Shear stress vs shear rate of pectin film forming solutions, measured at) °C. triangles are the experimental data, while the dotted lines represents the fitted curves based on Ostwald de Waele model
